# Aristotle's arm-swing hypothesis: biomechanical evidence from forward and inverse dynamics in an Olympic sprinter

**DOI:** 10.3389/fspor.2026.1845590

**Published:** 2026-06-24

**Authors:** Jiahua Li, Xiaoge Xiao, Hanyu Zhang, Manqi Ge, Long Chen, Wing-Kai Lam, Yifang Fan

**Affiliations:** 1 School of Physical Education and Sport Science, Fujian Normal University, Fuzhou, China; 2 School of Running, Fujian Normal University, Fuzhou, China; 3 School of Physical Education and Health, East China Normal University, Shanghai, China; 4 Department of Sports and Health Sciences, Academy of Wellness and Human Development, Faculty of Arts and Social Sciences, Hong Kong Baptist University, Hong Kong, China; 5 Centre for Exercise Science and Medicine (CESAME), Hong Kong Baptist University, Hong Kong, China

**Keywords:** arm swing, gait retraining, ground reaction force, running economy, running performance

## Abstract

**Introduction:**

Aristotle's observational hypothesis suggests that runners may run faster if they swing their arms, yet direct evidence that arm swings enhance propulsion during running is lacking. This paper aims to develop a non-VO₂-based computational pipeline to estimate propulsive and vertical impulses from pressure/force and kinematic data, and to examine whether arm-swing modification training can improve running performance.

**Methods:**

One world-class elite sprinter performed a laboratory-based arm-swing modification session on pressure treadmill. Kinematic and pressure data before and after modifications were collected by synchronized motion capture system and pressure treadmill system and analyzed with forward and inverse dynamics.

**Results:**

Results indicate that the linear displacement of the center of mass was highly consistent, and the integral changes of the Lagrangian were consistent with energy expenditure calculated by ACSM equations. The participant with modified arm swing exhibited smaller cadence, maximum ground reaction force, medial-lateral impulse integral, but increased step length and anterior-posterior and superior-inferior impulse integrals.

**Discussion:**

These results supported the observational hypothesis, which suggested that a short period of arm-swing modification training is practical to enhance running performance in laboratory settings. Moreover, digitalization, standardization, and program training protocols would enable coaches and practitioners to develop reliable interventions for improvements in running technique and athletic performance.

## 1 Introduction

Aristotle's observational hypothesis, suggesting that runners run faster if they swing their arms has been supported by biomechanists (1). Optimizing arm swing movement improves the center of mass (COM) stability, which is associated with better coordination of upper and lower limb movements for larger forward propulsion during running (2, 3). However, direct evidence that arm swings enhance propulsion during running is lacking (4). One possible reason is the reliance on VO₂ (gas-exchange)–based measurements of metabolic cost. Although laboratory “mixing-chamber” systems provide high accuracy, portable or breath-by-breath systems exhibit inter-system variability, require careful calibration, and show breath-by-breath fluctuations (5, 6), which reduces sensitivity to small or transient mechanical changes during gait (7). Another reason is that the high-dimensional problems associated with partial parametric differential equations remain unresolved, as noisy data is not yet reliably integrated into algorithmic frameworks (8). Insights from natural dynamic constraints and equilibrium help to accelerate the development of knowledge and technology (9) to examine whether arm swing contributes additional vertical impulse (i.e., lift) and forward propulsive effect (i.e., drive) during running (2). From a computational mechanics perspective, ground reaction forces (GRF) can provide a direct mechanical estimation for COM acceleration, displacement, and external mechanical work during locomotion (10, 11). Cavagna (10) showed that force-platform data can be used to estimate changes in total mechanical energy and positive external mechanical work during running. In addition, upper-limb motion may influence whole-body running mechanics by altering COM displacement, angular momentum balance, vertical impulse, and frontal-plane motion control (2, 3). These mechanical effects are closely related to training-relevant variables such as cadence, step length, step width, GRF, and impulse measures, because changes in stride frequency and stride length can alter COM vertical excursion, GRF, and lower-limb loading, while step width and arm swing are related to lateral balance during running (12, 13). Therefore, a computational approach combining pressure/force data, kinematics, and forward-inverse dynamics is needed to quantify how arm-swing modification may influence running biomechanics.

When the upper and lower limbs are simplified as chaotic pendulum models, their motion exhibits chaotic behavior (14). The motion of a chaotic pendulum, such as a double pendulum, is governed by coupled ordinary differential equations and is inherently chaotic (15). In this study, the lower limb is simplified as a chaotic pendulum model, from which the Lagrange equations of the human body are derived to evaluate running economy based on human complex system dynamics (16).

Previous studies have predominantly investigated the effects of natural and non-natural arm swing postures on metabolic and mechanical responses during running. Natural arm swing refers to the preferred arm swing as they could in their normal running condition, as it may vary across individuals, running speeds, and training background; whereas non-natural arm patterns refer to experimentally imposed arm position that restrict or exaggerate arm pattern such as placing both hands behind the back, hands on the head or swinging with larger range of motion (RoM) during running. From a metabolic perspective, Tseh et al. had nine female distance runners running at 3.35 m/s under four arm swing conditions: the natural arm swing, hands clasped behind the back, hands placed on the head, and exaggerated vertical oscillation conditions. Their results revealed that the participants significantly increased oxygen cost (VO_2_) when running with exaggerated vertical oscillation (≈ 51.0 mL·kg^−1^·min^−1^) and hands-on-head (≈ 46.1 mL·kg^−1^·min^−1^) conditions compared with the natural arm-swing condition (≈ 43.4 mL·kg^−1^·min^−1^), indicating that non-natural arm postures would impair running economy (17). Another study examined the metabolic cost during walking and running on a treadmill in each of three arm swing conditions: natural arm swing, arms crossed over the chest (no arm swing), and weighted elbows (higher segmental inertia); however, this study did not report a significant difference in metabolic cost between natural arm-swing and no arm-swing running, a finding explained by that the passive arm-swing motion's buffering of rotational forces (18). Arellano and Kram (19) further compared the physiological responses under natural arm-swing and no arm-swing conditions, including hands behind the back (BACK), arms crossed over the chest (CHEST), and hands on the head (HEAD) during treadmill running. Their findings indicated that compared with natural arm motion, non-natural arm positions required additional net metabolic power by approximately 3% (BACK), 9% (CHEST), and 13% (HEAD), respectively (19). However, the findings provide little insight into training concepts, as these arm positions are rarely used in realistic running conditions.

From a biomechanical perspective, previous treadmill running studies have shown that arm swing is related to lateral balance and upper-body rotational control during running. It is reported that restricting normal arm swing would increase metabolic demand, step-width variability, and compensatory shoulder and pelvis rotation (12, 19). More recently, forward-dynamics musculoskeletal simulation study treadmill running data showed that active arm swing would reduce torso angular motion and total metabolic energy consumption (20). Another overground running study revealed that suppressed arm swing was associated with reduced peak vertical GRF, increased peak lateral GRF, and altered lower-limb joint mechanics in frontal-plane motion (21). Although treadmill and overground running are generally biomechanically comparable, some differences have been acknowledged in sagittal-plane kinematics, contact time, stride length, and selected kinetic variables (22, 23).

Overall, these mechanical studies consistently highlight the role of natural arm swing in balancing angular momentum, stabilizing running posture, and reducing energy expenditure (4, 21). It is noted that the magnitude and nature of these effects depend on gender, training level, running speed (jogging vs. sprinting), measurement metrics (VO_2_, GRF, gait variability), and the specific arm-swing interventions employed. However, although all these metabolic and biomechanical studies have confirmed that natural arm swing outperformed unnatural arm swing, they did not explore how precise skill adjustments to swing amplitude, rhythm, or shoulder-elbow angles could translate into measurable speed improvement for real-world applications (24, 25). Moreover, most of the studies considered arm swing in isolation, but did not consider its synergy with other key training-related elements including stride frequency, stride length, and step width, and thus failed to provide practical guidelines and strategies for improving athletic performance (19, 26). This emphasizes that arm swing is part of the whole-body locomotor system rather than an isolated movement component. Adopting the arm swing technique from elite athletes may have potential to improve gait mechanics and running economy of experienced runners.

This study emphasizes a non-VO₂-based approach: we developed a computational pipeline to estimate propulsive and vertical impulses from pressure/force data and kinematics, and then we validated the feasibility through an arm-swing modification training case. The purpose of this single-participant exploratory study was to examine the arm-swing contribution to propulsive and vertical impulses and to assess whether the elite-specific arm-swing modification training enhances running gait and economy. We hypothesized that the modified arm-swing technique would be associated with higher propulsive and vertical impulses, and better estimated running economy, compared with the natural arm-swing condition. The findings of this study may provide preliminary and feasibility-based insights into how specific arm-swing techniques could contribute to better running mechanics and estimated running economy.

## 2 Methods

### 2.1 Participant

Although individual runners have vary anthropometric characteristics and movement preferences, the fundamental biomechanical principles underlying technique optimization are broadly applicable (27), which allows gait training in rehabilitation and athletic training. In this exploratory study, we have recruited one Olympic-level sprinter as it is challenging to recruit multiple Olympic-level sprinters for an individualized technique-modification intervention such as the time and location limitation. Regarding scientific merit, single-subject studies are also considered useful in applied sport science because they allow researchers to examine how a specific training or conditioning intervention works for a particular athlete rather than for an averaged group response (28). For example, Chen et al. (29) reported a potential application stepwise high-intensity training intervention in a former world-champion badminton player with knee pain, which showed that the athletes reduced knee pain symptoms and improved agility-task performance and cardiopulmonary fitness after three-week training and returned to regular training immediately and participated international competitions within one month (29). Furthermore, highly skilled athletes generally exhibit more consistent movement control than lower-skilled athletes, which may improve the interpretability of athlete-specific biomechanical responses (30). One female sprinter (age: 24 yrs; height: 163 cm; weight: 57 kg) was recruited for this study. The participant has been a member of the Chinese national team since 2012 and has competed in three consecutive Olympic Games. To improve her arm-swing technique for better sprint performance, both the participant and her head coach agreed to participate in this study. The participant had no injuries to the upper limbs, lower back, or lower limbs in the past six months before data collection. The experimental procedure was in accordance with the Declaration of Helsinki and approved by the Ethics Review Committee of Fujian Normal University. Informed consent was obtained from the participant prior to the experiment.

### 2.2 Selection of technical model

Drawing on the research team's extensive experience working with elite Olympic athletes, the arm-swing pattern of American World Champion Jeremy Wariner was selected as our technical framework for this study. Wariner was prioritized for three reasons: (1) His distinguished competitive career, including four Olympic medals and a running form widely recognized in the biomechanics community for its exceptional fluidity and efficiency (31); (2) his running video and related biomechanical information allowed quantitative analysis of arm–trunk coordination, including the right upper-limb COM position relative to the shoulder joint, upper-arm swing range, and the linear displacement, linear velocity, and linear impulse of the upper-limb segments and hand relative to the shoulder joint; and (3) His coach, Clyde Hart, is renowned in track and field community for emphasizing data driven optimization via biomechanics over intuitive methodologies (32), aligning with the scientific rigor of this study. Therefore, Wariner's arm-swing pattern was used as an elite technical reference aiming to improve running performance of our Olympic participant.

### 2.3 Development of the quantitative model

To establish the training protocol, a senior biomechanist and a head track and field coach reviewed Wariner's video and calculated the position of his right upper-limb COM, which remained in the third quadrant of a Cartesian coordinate system with the shoulder joint defined as the pivot point, as shown in Supplementary Figure S1. Wariner's running video was obtained from a publicly available YouTube source, and original biomechanical data granted by Dr. Peter Weyand to the corresponding author via email. The video and related biomechanical information were collected under treadmill-running conditions at an approximate speed of 9.09 m/s. The biomechanical data presented in the video were digitized and processed using OriginPro. Forward-dynamics calculations were subsequently applied to estimate Wariner's COM acceleration, velocity, displacement, kinetic energy, and Lagrangian-related variables, as detailed formula and procedure are provided in Supplementary Figures S2–S7. Next, Wariner's arm-swing motion was deconstructed from two perspectives: (1) Point-mass system: In a sagittal-plane Cartesian coordinate system, the shoulder joint was defined as the origin, the positive *x*-axis directed anteriorly and the positive *y*-axis directed superiorly, the upper-limb COM remained predominantly in the third quadrant throughout the arm-swing cycle (Supplementary Figure S8); (2) Rigid-body system: The upper arm was defined by the acromion process and the lateral epicondyle of the humerus. The upper arm remained in the third quadrant, swinging nearly 180° behind the shoulder and no more than 270° in front, with the forward swing close to the acromion process. Based on Wariner's running technique observed in the video, upper-limb inertial parameters were used to calculate the linear displacement, linear velocity, and linear impulse of the upper-limb segments and hand relative to the shoulder joint during running (Supplementary Figures S2–S7). Finally, an elite-specific arm-swing modification protocol was developed by incorporating Rabita et al.'s (33) acceleration feedback methods alongside findings by Arellano & Kram (19) and Pontzer et al. (18) regarding metabolic cost and postural stability (18, 19, 33).

### 2.4 Biomechanical analysis and optimization protocol

The experimental protocol included warm-up, treadmill habituation, pre-test, arm-swing modification training, and post-test procedures, as detailed in Section 2.6. Skill optimization protocols using multidimensional data and real-time feedback are commonly used to enhance athletic performance (34, 35). To ensure the accuracy and reliability of the sagittal-plane COM trajectory during performance optimization, this study applied both forward dynamics (10) and inverse dynamics (1) as complementary approaches to calculate the average COM trajectory. The marker-based inverse-dynamics approach was used to estimate sagittal-plane segmental COM positions and the whole-body COM trajectory from kinematic data and anthropometric parameters (36, 37). The plantar-pressure-based forward-dynamics approach was used to derive GRF-related COM acceleration, velocity, displacement, kinetic energy, and impulse-related variables (10, 11, 37). This combined approach was used to cross-check kinematic and kinetic estimates of COM motion rather than to replace conventional marker-based COM estimation, because forward dynamics estimates body COM motion from GRF, whereas inverse dynamics estimates COM position and velocity from body-segment motion (37, 38).

**Figure 1:**
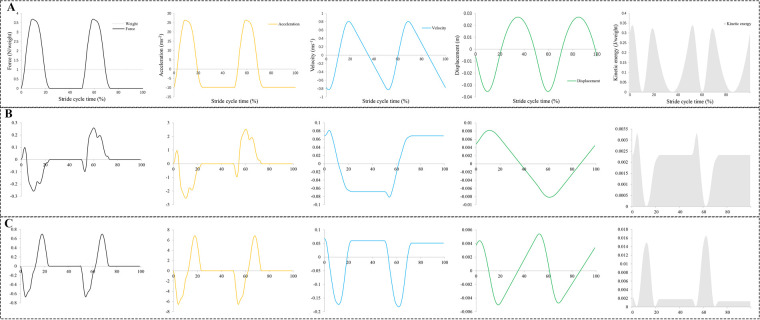
World champion data of jeremy wariner derived from 3D treadmill and forward kinematics analysis of COM. Acceleration, velocity, displacement, kinetic energy, and Lagrangian of the COM in **(A)** superior-inferior direction, **(B)** medial-lateral direction, and **(C)** anterior-posterior direction.

To further explore the geometric relationships among COM acceleration, velocity, displacement, kinetic energy, and Lagrangian equations-related variables, GRF data were normalized to the corresponding runner's body mass. Specifically, Wariner's biomechanical data were normalized to Wariner's body mass, whereas the GRF data obtained from the participant on the instrumented treadmill (FDM-THQ-M-3i, zebris Medical GmbH, Germany) were normalized to the participant's body mass. A forward-dynamics approach was then applied to establish a quantitative model for arm-swing training and analysis, including arm–trunk coordination during running (Figure 1). Drawing on the research team's cumulative training and biomechanical analysis experience, the sagittal plane COM trajectory was derived from both forward and inverse dynamics, and therefore running economy was subsequently quantified exclusively using kinetic data from the pressure treadmill. For the elite-specific arm-swing technique (Table 1), the key movement elements were developed for the backward and forward swing phases and were confirmed by the senior biomechanist and head coach. These elements were described using observable biomechanical characteristics, including arm-trunk coordination, trunk stability, and avoidance of excessive vertical oscillation. Nevertheless, because this was an exploratory single-participant study, fixed quantitative movement thresholds were not predefined. Future studies should establish objective biomechanical criteria and quantifiable thresholds to enhance reproducibility. The backward swing movements allowed for maximum counter-rotation torque against the driving leg (i.e., opposite front leg), while the forward swing movements maximized trunk stability and minimized unnecessary vertical oscillation.

**Table 1:** Instructions for elite-specific arm-swing technique.

Arm swing	Instructions	Illustration
Backward	Upper arm extends smoothly behind the trunk, approaching a full 180° relative to the shoulder axis. At the maximum posterior, the humerus lies nearly horizontal with minimal elbow flexion. This backward movement reaches maximal to generate counter-rotation torque against the driving leg (i.e., opposite front leg).	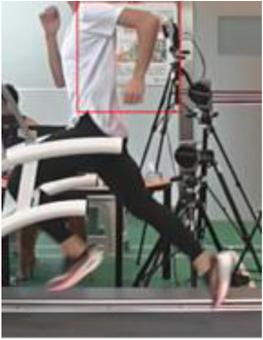
Forward	Upper arm accelerates forward to no more than 270° in the sagittal plane. Hand moves close to the deltoid insertion of the shoulder. This controls foward excursion. The arm's COM is ensured to remain within the third quadrant.	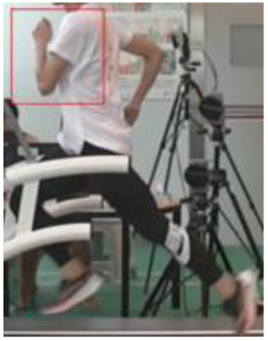

### 2.5 Equipment

The motion capture system of 18 Oqus700+ infrared cameras (Qualisys AB, Sweden, sampling at 1,500 Hz) was used to collect marker trajectories for estimating segmental COM positions and whole-body kinematics running at 3 m/s on the pressure treadmill. For our training and assessment setup, the capture volume was 3 × 1.8 m and the measurement error was approximately 0.2 cm, ensuring data accuracy within a 1 cm threshold. The pressure treadmill with a sampling frequency of 300 Hz was used to measure plantar pressure and spatiotemporal data.

### 2.6 Procedure

The participant reported to the laboratory to complete a standardized technical learning protocol. The participant repeatedly watched the same video of Jeremy Wariner's running mechanics that was developed the model in Section 2.2. In addition, the participant further studied the key running phrases illustrating arm–trunk coordination, as shown in Supplementary Figure S1. Then the participant was provided with written instructions and was instructed on each of the key elements of arm-swing modifications (Table 1). Prior to data collection, the participant completed a 10 min structured warm-up including a 3 min jogging, 4 min dynamic stretching, and 3 repetitions of three 30-m accelerations. To minimize errors between the markers and the actual joint centers, eleven reflective markers were attached directly over the right side of the participant's skin (and shoe), including the head of the fifth metatarsal, the head of the first metatarsal, the lateral malleolus, the calcaneus, the lateral femoral epicondyle, the greater trochanter, the acromion process, the lateral epicondyle of the humerus, the fifth metacarpophalangeal joint, the radial styloid process, and the ulnar styloid process. To ensure accuracy, the simple marker set was used to define a sagittal-plane linked-segment model of the right lower limb, trunk, and upper limb, rather than to reconstruct a full three-dimensional whole-body COM model. This marker configuration was to estimate sagittal-plane segmental COM changes and arm-trunk coordination only, rather than to calculate full three-dimensional joint angles. Our simple marker set was selected to reduce experimental complexity and minimize potential interference with natural running movement. The selected anatomical landmarks represented the major segment endpoints required for two-dimensional geometric and inverse-dynamics analyses, which commonly rely on segmental kinematics, anthropometric parameters, and external force data to estimate body-segment and joint-level dynamics (36, 39). In the present study, whole-body COM-related variables were primarily derived from the GRF measured by the instrumented treadmill using a forward-dynamics approach, based on the mechanical relationship between external force and COM acceleration (11, 40). Therefore, the 11-marker set was considered appropriate for the sagittal-plane arm-trunk coordination and linked-segment analyses conducted in this study.

The participant then performed a 3 min running with her regular training shoes on the pressure treadmill at 3 m/s to adapt the treadmill system, followed by a 3 min rest. After treadmill running familiarization, the participant completed a 3 min pre-test running trial at 3 m/s under her natural arm-swing condition. During the training session, the participant performed three 2 min arm-swing modification sets at 3 m/s, with 5 min rest intervals between sets, with reference to the Wariner arm-swing pattern (Supplementary Figure S1 and Table 1). Real-time verbal cues and instructions were provided by experimenters to help the participant acquire the new arm-swing pattern. After the training sessions, the participant's modified arm-swing technique was evaluated by the senior biomechanist and head coach based on the key movement elements described in Table 1, including arm–trunk coordination during the backward and forward swing phases. Once the participant was able to consistently perform these movement elements during the final training set, she completed another 3 min post-test running trial to measure arm-swing angle, peak GRF, cadence, and step length for assessing the training effect of arm-swing technique. After data collection, COM kinematics (3) was used to calculate the positional coordinates of each body segment and the whole body relative to the inertial reference frame. Specifically, segmental COM positions were estimated from the anatomical landmark coordinates and anthropometric parameters, and the whole-body COM was calculated as the mass-weighted sum of the segmental COM positions. The upper limb is provided as an example of this calculation in Supplementary Figure S8.

### 2.7 Data processing

In accordance with the complementary forward- and inverse-dynamics approach described above, marker-based inverse dynamics was used to calculate segmental COM positions and the whole-body COM trajectory from joint-marker coordinates and anthropometric parameters. Segmental COM velocity and acceleration were obtained from the first- and second-time derivatives of COM position, respectively. Plantar-pressure-based forward dynamics was used to derive GRF-related COM acceleration, velocity, displacement, kinetic energy, impulse-related variables, and Lagrangian-related variables from the instrumented treadmill data. These two approaches provided complementary kinematic and kinetic estimates of COM motion rather than being merged into a single COM model. The resulting COM and GRF-related time-series data were normalized to the gait cycle and processed using Fourier-transform-based denoising before the calculation of normalized impulse and Lagrangian integrals.

Inverse dynamics was applied to obtain the segment COM positions through joint markers of the participant, as described in the previous study (41). Human body segment inertial parameters including segment geometric dimensions, mass, and moments of inertia were used to analyze the kinematic and dynamic characteristics of the segments of the runners (36). Specifically, the position of upper arm segment COM was calculated using the shoulder and elbow joint markers with the inertial parameters of the upper arm; the position of forearm segment COM was calculated using elbow and wrist joint markers with the inertial parameters; the hand marker position was directly represented as the hand segment COM position. For the lower extremity, the position of thigh segment COM was calculated using the hip and knee joint markers with the inertial parameters of thigh; the calf segment COM positions was calculated using knee and ankle joint markers with the inertial parameters; the foot COM position was calculated using markers of the heel, the first and the fifth metatarsophalangeal joints; the head and trunk COM positions were calculated via head and trunk markers with their respective inertial parameters.

The absolute COM velocity and acceleration of the segment relative to inertial reference frame were determined by the first- and second-time derivatives of the COM position, respectively. Finally, we used the shoulder and hip joints as the rotation points for the upper and lower limbs, respectively, and a Cartesian coordinate system was constructed in the sagittal plane. The angular displacement of the segments was calculated based on the change of segmental COM position in the sagittal plane. The upper and lower limb segment inertial parameters were used to calculate the centrifugal force of the segment to obtain the kinematic parameters, including linear and angular acceleration, velocity, and displacement.

To overcome the potential errors due to inverse dynamics approach (e.g., sensor errors and assumptions) (38, 42, 43), additional forward dynamics calculation was applied to adjust the model for this study. The forward dynamics method is highly reliable for calculating the COM dynamics in running biomechanics (44–46). Forward dynamics simulations were employed to quantify the contribution of GRF during running (47). The same training method has been used for a world-class sprinter, Matt Bundle from the University of Montana to assess the training progression (48). The forward dynamics based on plantar pressure data was used to measure the kinematics and kinetics of the whole body COM as described in the previous study (16) with the following procedures:

#### 2.7.1 Lagrangian equation

Both upper and lower limbs were simplified into two triple-pendulum structures which comprised the hand, forearm, and upper arm and the foot, calf, and thigh, respectively. The Lagrangian equations for the upper and lower limbs were then used to calculate the energy expenditure by the muscle within a gait cycle. See more details in Supplementary Appendix A. It should be noted that although previous studies have shown associations between mechanical work/energy variables and metabolic energy expenditure, the relationship between these variables requires further investigation. Future studies should further validate this approach using physiological or metabolic measurements, such as VO₂ analysis.

#### 2.7.2 Data denoising

The Euler formula was used to describe human COM motion in the horizontal and vertical planes (49). The COM data were normalized with the gait cycle. The noise due to human segment marker was filtered by Fourier Transform and the Fourier series expansion formula were determined using the Least Square Fitting Methods (50). The regression matrix of least squares fitting was constructed to unify the constant, sine, and cosine terms into a matrix to facilitate linear regression (51–53). The root mean square deviation was determined and approximate calculation of curvature by first-order and second-order differentiation was applied to residuals to analyze their angular velocity and acceleration of fitting precision. See more details in Supplementary Appendix B.

By jointly modeling the anatomical joint constraints (e.g., range of motion, mechanical equilibrium) with the objective of minimizing soft tissue artifact, a more comprehensive and optimal framework was constructed to optimize soft tissue artifact compensation, and a more reliable solution was achieved for our study (54). The product of the normalized force and the percentage of stride time was referred to as the normalized impulse. The integration of the standardized impulse by the natural and modified arm-swing patterns are provided in Table 2. In addition, the forward dynamics method (55) was used to calculate the integral of the Lagrangian over stride cycles.

**Table 2:** Normalized impulse integral of the arms, legs, and body per running cycle between natural and modified arm swing.

Arm Swing		Arms			Legs			Body		
		*x*	*y*	*z*	*x*	*y*	*z*	*x*	*y*	*z*
Natural	R	−1.10	0.05	−7.71	−4.17	0.01	−42.89	−5.28	0.05	−49.54
	L	−0.92	0.06	−9.28	−4.94	0.00	−41.77	−5.86	0.06	−50.12
Modified	R	−1.48	0.03	−8.17	−9.58	0.01	−44.31	−11.00	0.03	−51.54
	L	−1.33	0.05	−8.57	−10.73	−0.01	−44.05	−12.01	0.04	−51.96

### 2.8 Data analysis

**Figure 2:**
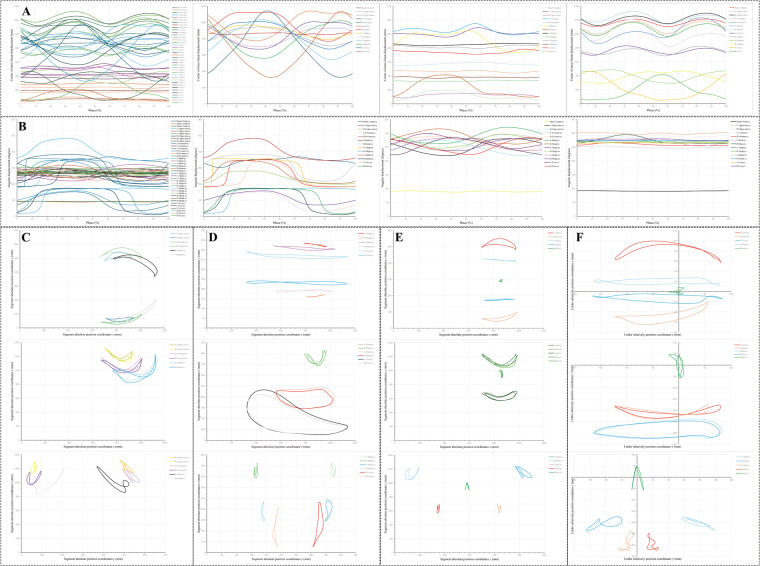
Generalized displacement of human segments, limbs and whole body. **(A)** Linear displacement and **(B)** angular displacement of limbs and whole body relative to the inertial system, respectively; Movement path of **(C)** upper limb and **(D)** lower limb; **(E)** Movement path of four limbs and whole body; **(F)** Movement path of four limbs and whole body when upper limbs are relative to shoulder joint and lower limbs relative to hip joint.

First, generalized displacement trajectories of the human segments, limbs, and whole body were calculated using forward- and inverse-dynamics approaches to describe sagittal-plane movement patterns during running (Figure 2). Second, the linear and angular velocities of the joints relative to the inertial reference frame were used to calculate the translational and rotational kinetic energy. Although the sagittal-plane COM coordinates fluctuated during running, the net integral of COM displacement over the normalized gait cycle was close to zero, as shown in Supplementary Figures S5, S6. Therefore, the absolute value of the changes in the segment Lagrangian was used to determine the work done by muscles in an average of 15 stride cycles, which is indicative of calories per hour consumed by the participant during running.

## 3 Results

### 3.1 Waveform comparison and arm-swing contribution on GRF impulses

**Figure 3:**
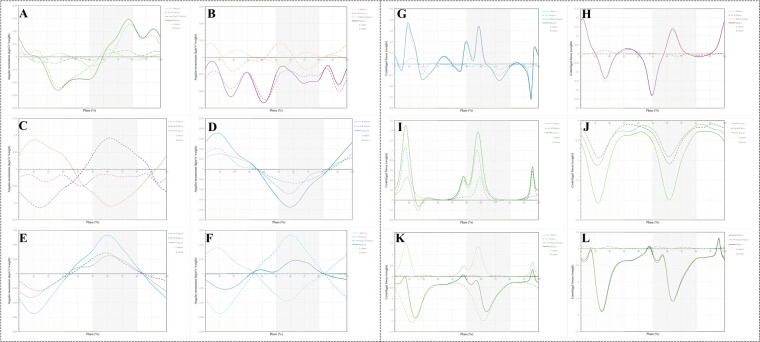
Upper limb function during running (3 m/s)—angular momentum and centrifugal force with a-f for natural arm swing and g-l for modified arm-swing. Angular momentum of upper limbs, head and trunk, lower limbs and the whole body in **(A)** coronal plane and **(B)** sagittal plane; **(C)** Angular momentum of left leg, right leg and the lower limbs in sagittal plane; Angular momentum of left arm, right arm and upper limbs in **(D)** transverse plane and **(E)** frontal plane; **(F)** Angular momentum of upper limbs, head and trunk, legs and the whole body transverse plane; Centrifugal force of upper limbs, head and trunk, lower limbs and the whole body in **(G)** anterior-posterior and **(H)** medial-lateral directions; **(I)** Centrifugal force of left arm, right arm, upper limbs in superior-inferior direction; **(J)** Centrifugal force of left leg, right leg and lower limbs in superior-inferior direction; **(K)** Centrifugal force of upper limbs, head and trunk, lower limbs and the whole body in superior-inferior direction; **(L)** Centrifugal force of four limbs, head and trunk and the whole body in superior-inferior direction. The shadowed, light and deeper areas refer to stance, right stance, and left stance phases, respectively.

**Figure 4:**
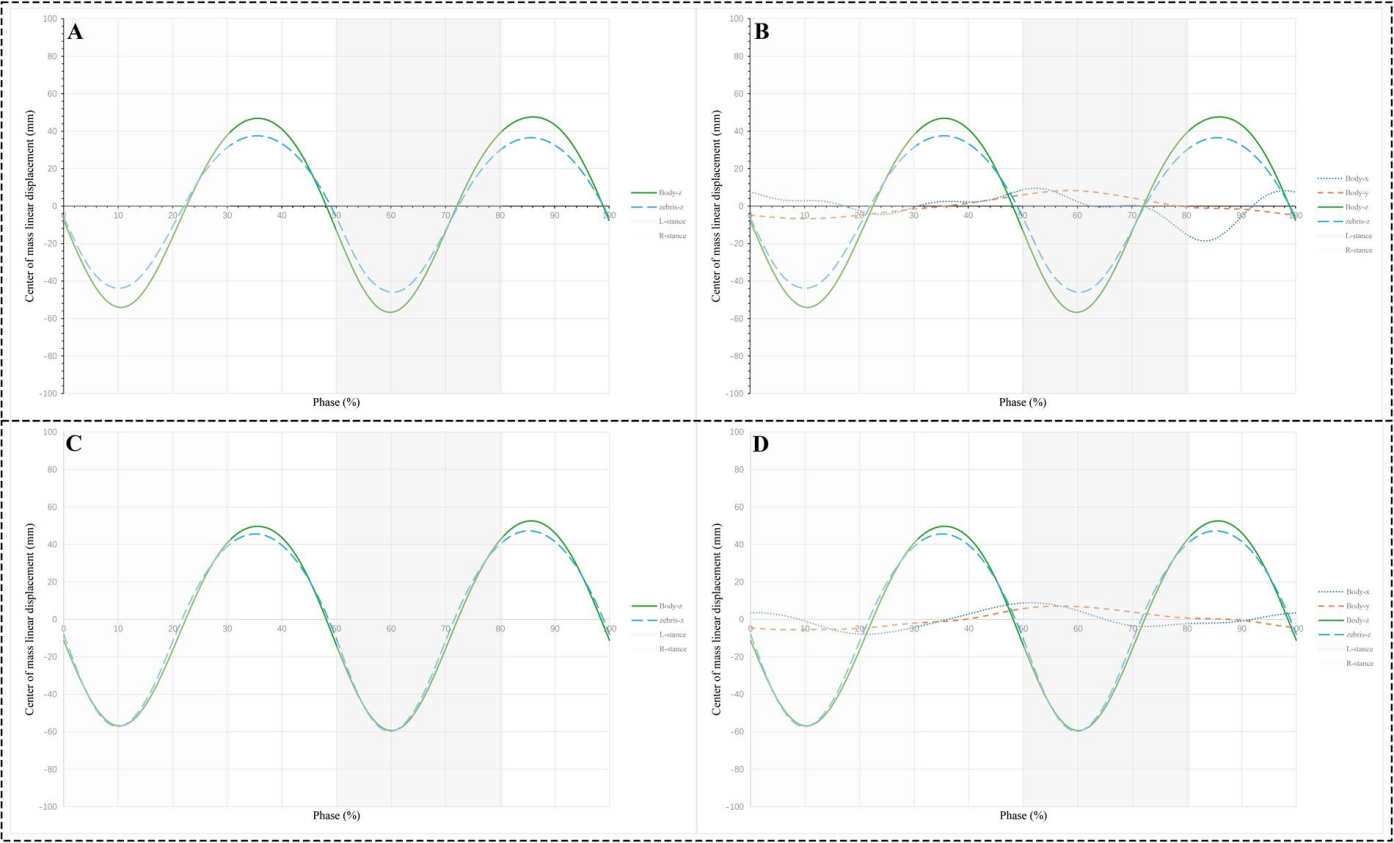
COM linear displacement of the average stride cycle (3 m/s). Linear displacement of the whole body in **(A)** superior-inferior direction and **(B)** anterior-posterior, medial-lateral, and superior-inferior directions when running in natural arm swing; Linear displacement of the whole body in **(C)** superior-inferior direction and **(D)** anterior-posterior, medial-lateral, and superior-inferior directions when running in controlled arm swing. The shadowed, light, deeper areas refer to the stance, right stance, and left stance phases, respectively.

**Figure 5:**
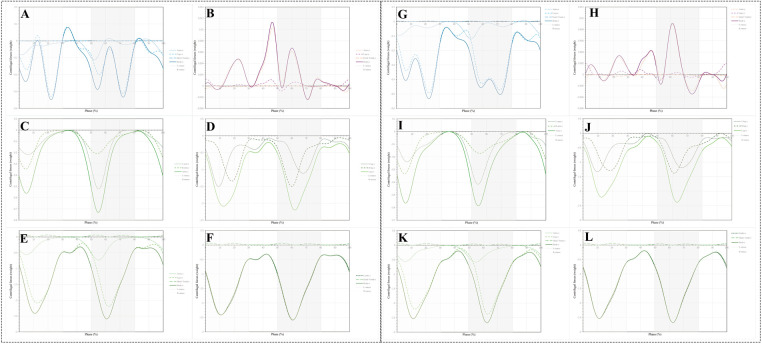
Four limb function during running (3 m/s) with a-f for natural arm swing and g-l for modified arm-swing. Centrifugal force of the upper limbs, head and trunk, lower limbs and the whole body in **(A)** anterior-posterior direction and **(B)** medial-lateral direction; Centrifugal force of the **(C)** upper limbs and **(D)** lower limbs in superior-inferior direction; **(E)** Centrifugal force of the upper limbs, head and trunk, lower limbs and whole body in superior-inferior direction; **(F)** Centrifugal force of four limbs, head and trunk, and the whole body in superior-inferior direction; Centrifugal force of upper limbs, head and trunk, lower limbs and the whole body in **(G)** anterior-posterior direction and **(H)** medial-lateral direction; Centrifugal force of **(I)** upper limbs and **(J)** lower limbs in superior-inferior direction; **(K)** Centrifugal force of upper limbs, head and trunk, lower limbs and the whole body in superior-inferior direction; **(L)** Centrifugal force of four limbs, head and trunk, and the whole body in superior-inferior direction. The shadowed, light, deeper areas refer to stance, right stance, and left stance phases, respectively.

A comparative analysis (Figure 3) revealed that our waveform patterns were similar to those from Hinrichs' study (2), entitled “Upper extremity function in running. II: Angular momentum considerations”. Specifically, our Figures 3A–F correspond closely to Hinrichs' Figure 3, 4, 5, 7, 8, 10, and 11, respectively (2). Figure 4 shows that the change of COM from pressure data in the superior-inferior direction was generally smaller than that obtained from the kinematics data, which suggests that pressure data would underestimate the peak vertical GRF (56). In addition, the angular momentum of the upper limbs (Figure 3B) was more consistent with the findings of 10 sprinters from Froidmont's study (57). However, according to Newton's Third Law of Motion, Figures 3I,K suggest that in the stance phase during running, the upper limbs did not show any obvious increase in lift (vertical impulse), which is inconsistent with findings from the studies of Hinrichs and Froidmont (1, 3, 57).

Given that the arm and leg rotated about the shoulder and hip joints, respectively, the participant was trained with the arm-swing modification techniques on a pressure treadmill. The centrifugal forces of the upper limbs, head and trunk, lower limbs and the whole body were calculated and shown in Figure 5. The upper limbs' contributions in Figures 5C,E,I,K are consistent with the findings from Hinrichs and Froidmont (1, 3, 57). Figure 5A shows that the arm swing would generate forward propulsion except during the right stance phase, and the force of the lower limb in the anterior-posterior direction was positive during the left stance phase. Supplementary Figure S9 shows that the modified arm swing contributed 12.21% to the drive and 16.17% to the lift at a running speed of 3 m/s.

### 3.2 Feasibility of energy expenditure determined by muscle work

The energy expenditure estimated from muscle work within stride cycles (Other Supplementary Data Test Reports S1, S2) was 654.47 cal/h and 616.16 cal/h for natural and modified arm swing, respectively. These values should be interpreted as mechanically estimated energy expenditure rather than direct physiological or metabolic measurements. For comparison, energy expenditure was also estimated using physiological equations (58, 59, 88). See more details in Supplementary Appendix C. In addition, the forward dynamics method (55) was applied to calculate the integrals of the Lagrangian over two normalized stride cycles, which were 16.59 J (weight) and 22.16 J (weight).

### 3.3 Efficacy of the elite-specific arm-swing training

The centrifugal forces of the whole body in the natural arm swing (Figure 5F) and modified arm swing (Figure 5L) were generally similar to those in the superior and inferior directions (Other Supplementary Data Test Reports S1, S2), respectively. Figures 5A,G showed that the COM of both upper and lower limbs was located in Quadrant III in the sagittal plane during running.

Moreover, the participant with modified arm swing exhibited smaller cadence (−4.3%), peak vertical GRF (−2.3 to −3.0%), medial-lateral impulse integral (−36.4%), but increased step length (3.6%) and anterior-posterior (106.6%) and superior-inferior (3.85%) impulse integrals. See more details in Table 2, Supplementary Figure S9, and Other Supplementary Data Test Reports S1, S2. Moreover, the estimated energy expenditure was lower in the modified arm-swing condition compared with that in the natural arm-swing condition (Table 3).

**Table 3:** Work done by the muscle per running cycle between natural and modified arm swing.

Arm swing	Translation work (J/weight)				Rotation work (J/weight)				Sum (J)	
	*x*	*y*	*z*	sum	*xy*	*xz*	*yz*	sum	work	calories
Natural	2.77	0.12	4.52	7.41	2.00	2.64	0.09	4.73	563.12	0.1345
Modified	2.61	0.14	4.54	7.29	1.81	2.74	0.12	4.66	551.65	0.1318

Sum of translation and rotation work (translation in the superior-inferior direction offsets work done by gravity); overall calories expressed in kcal. The *x*, *y*, and *z* denote the work in medial-lateral, anterior-posterior, and vertical direction, respectively, while *xy*, *yz*, and *xz* denote the rotational work about *z*-axis, *y*-axis and *x*-axis respectively.

## 4 Discussion

This study examined the arm-swing contribution to GRF impulse and the efficacy of elite-specific arm-swing modification training in an Olympic sprinter. In the real-world athletic skill modification training, it is difficult to measure the running economy with physiological VO_2_ devices, as the athletes are required to wear the additional weight of the device and face-mask, which may influence the actual assessment of skill modification (60). To verify Aristotle's arm-swing hypothesis, we conducted the first study to apply both inverse and forward dynamics to develop an arm-swing modification training protocol and its mechanical model to monitor the GRF and running economy before and after training. A key benefit of our proposed mechanical model is that it allows continuous skill modifications feedback based on GRF and gait parameters (61–65), with minimal interference to natural running.

Our results were consistent with previous studies by Hinrichs and his colleagues (1–3). The centrifugal-force analysis of arm-swing data showed that the participant generated smaller vertical and forward force components after the elite-specific arm-swing modification. This finding partly supported Aristotle's observational hypothesis that arm swing influences running performance. Specifically, at the same running speed, step width decreased from 9 cm to 7 cm, stride length increased from 2.22 m to 2.30 m, and cadence decreased from 163 steps/min to 156 steps/min after the technical modification. These changes suggest that the modified arm-swing pattern was associated with lower upper-limb swing frequency and longer stride pattern. Since centrifugal force is proportional to the decrease in upper-limb angular velocity may partly explain the reduced vertical GRF component observed after modification, as shown in Other Supplementary Data Test Reports S1, S2. Furthermore, the anteroposterior centrifugal force of the upper limb was directed predominantly backward during the stance phase in Figure 5A and consistently backward throughout the stance phase in Figure 5G. Based on the action–reaction principle, this backward-directed upper-limb force may be related to a forward-directed reaction within the whole-body system during stance. Therefore, the observed reductions in lift and drive should not be solely explained by a simple decrease in arm-swing contribution. Instead, arm-swing modification was showed to redistribute force distribution, COM dynamics, and gait spatiotemporal characteristics during running.

Based on inverse dynamics (41), segment and joint center data were used to calculate segment COM positions and kinematic parameters, including linear velocity and acceleration, and angular acceleration of the limbs (66–68). However, the analysis of COM would induce non-physical residual forces/torques for model compensation (69), which reduces the accuracy of the analysis (38, 43). To address this issue, we applied a denoising algorithm based on Fourier fitting and adaptive reconstruction to process the Qualisys marker trajectories. This approach not only preserved the essential characteristics of the raw kinematic signals but also improved the robustness and reliability of inverse and forward dynamic calculations, as indicated by the reduction of noise-related artifacts and residuals. Forward dynamics offers a highly reliable approach for calculating COM dynamics during running (44–46), effectively complementing and addressing the inherent limitations of inverse dynamics. Furthermore, forward-dynamics models driven by plantar pressure data enable the estimation of GRF, facilitating a comprehensive analysis of both the kinematics and kinetics of the whole body COM (70, 71). Accordingly, incorporating forward dynamics into inverse dynamics, combined with an enhanced preprocessing pipeline, was applied to minimize calculation errors and improve the physical plausibility of computed forces and torques. Moreover, integrating this computational pipeline into a digital platform would enable the design of tailored training interventions. Such a system would be rapidly deployed and iteratively optimized for individual athletes, accelerating improvements in running technique and competitive performance.

Arm swing can be approximated as a pendulum-like motion, where oscillation frequency is mainly determined by the effective length of the arm and gravitational acceleration (72, 87). Previous studies have suggested that arm swing can partly function as a passive mass-damping mechanism during walking and running that reduces upper-body rotation, while rhythmic arm and leg movements are also regulated by central pattern generator activity and sensory feedback (18, 73, 74). Therefore, in a well-practiced and stable running pattern, arm swing may show pendulum-like regularity, whereas its amplitude, timing, and coordination remain actively regulated by the neuromuscular system (75). According to Stokes' theory (76), the integral of the centrifugal force in the medial–lateral direction is approximately zero due to the symmetric nature of pendulum-like motion, positive and negative displacements tend to cancel each other out. As such, arm swing has a minimal contribution to drive force, which is consistent with previous studies e.g., (57) Furthermore, since the upper limbs have relatively lower segmental mass of approximately 4.94% compared with the trunk and lower limbs (36), the upper arms would have a smaller contribution to lift force (77). During running, although arm swing has been shown to contribute minimally to drive force, it plays an important role in maintaining postural stability and dynamic balance (78). However, the data from our Olympic sprinter indicated that, during constant-speed running, arm swing contributed substantially to lift impulse but negligibly to drive impulse (57, 78). In the present study, Figures 5A,G showed that, during the stance phase, the anteroposterior centrifugal force of the upper limb was directed predominantly backward, suggesting that the upper limb may contribute to a forward reaction through the trunk–stance-limb–support-surface linkage. In addition, Figures 5E,K showed that the vertical component of upper-limb centrifugal force varied during the stance phase, indicating that the upper limb may also contribute to the vertical support reaction through the same whole-body mechanical linkage. These findings suggest that sprint arm swing cannot be regarded as a purely passive pendulum motion; instead, it should be considered as an actively controlled and coordinative component of whole-body running mechanics.

After few arm-swing training, our Olympic sprinter showed changes in running biomechanics, including lower cadence, lower peak vertical GRF, and a reduced medial-lateral impulse integral, together with increased step length and anterior-posterior and superior-inferior impulse integrals. These preliminary findings suggest that running arm-swing technique may be acquired and trained through short-term training. In the future, randomized controlled studies are needed before a viable conclusion can be made for broader running populations. Moreover, skill-optimization platforms with on-line visual feedback can offer objective, data-driven insights into performance metrics. In addition to basic gait parameters such as step width, step length, and running speed, it is also important to normalize segmental and whole-body impulses to body weight, such that the impulse contributions of different body segments can be compared across running conditions. This approach is methodologically consistent with previous studies that used normalized upper-limb angular momentum to evaluate the contribution of arm swing to whole-body running mechanics (4). The previous studies (3) revealed that the arm swing contribution was approximately 5%–10% to the total vertical impulse (lift), the trunk momentum contributed a net decrease (−3%) to the total vertical impulse, while the legs contributed about 98% to the total lift. These results suggest that arm swing contributes minimally to lifting forces in running. Consequently, caution is warranted when recommending specific arm-swing techniques in running for performance enhancement (27). Considering many confounding factors for running performance (79, 80), studying biomechanics, physiology and data science may help optimize running techniques and performance (81).

In this study, Lagrangian mechanics provided a useful framework for analyzing dynamic systems and estimating energy expenditure during running from a biomechanical perspective. The results were similar to those commonly calculated using physiological equations (58, 59, 82–84). This provides a biomechanical perspective by which Lagrangian dynamic equations may help elucidate the underlying mechanisms of complex physical systems (85).

Several limitations should be considered when interpreting the findings of this study. First, this is an exploratory pilot study using available elite technical reference data to examine the feasibility of individualized arm-swing modification training. Analyzing the current world-record holder (Wayde van Niekerk) or more recent elite sprinters (Quincy Hall or Matthew Hudson-Smith) would provide a valuable direction for future research. Second, only one Olympic female sprinter was trained with the elite-specific arm-swing modification session. Our results may not be generalizable to lower-level amateur runners, as the arm-swing modification alone may not be effective to enhance overall running performance due to smaller arm-swing contribution than the contributions from trunk or lower limbs. In addition, this study was conducted on a pressure treadmill at 3.0 m/s with only one participant, it remains unclear whether the findings can be transferred to overground sprinting, acceleration, or competition-specific running conditions. Future validation in overground and track-based settings is needed before a viable conclusion can be made. Second, although our training has been used for individual Olympic athletes, future randomized controlled trials involving amateur athletes should be conducted to develop a general guideline for arm-swing technique in running. Third, the algorithm and validation in our study were based on the data extracted from instrumented pressure treadmill, new algorithm and validation should be developed when the instrumented three-dimensional force plate treadmill is used in other laboratory settings. Finally, only the simplified marker set was used to estimate COM and model calculation but cannot fully capture three-dimensional whole-body motion or transverse- and frontal-plane segment rotations. Future studies should employ a more comprehensive full-body marker configuration or three-dimensional motion-capture protocol to improve the validity and accuracy of whole-body biomechanical modeling (86).

## 5 Conclusions

By systematically adjusting arm mechanics, the participant achieved measurable improvements in speed and running efficiency, leading empirically support to Aristotle's observational hypothesis that runners can run faster when they swing their arms. Future study should explore the underlying biomechanical and neuromuscular mechanisms in real-world running contexts.

## Data Availability

The original contributions presented in the study are included in the article/Supplementary Material, further inquiries can be directed to the corresponding author/s.
