# Supplementary material for: Aristotle's arm-swing hypothesis: biomechanical evidence from forward and inverse dynamics in an Olympic sprinter
Source: Front Sports Act Living. 2026 Jun 24;8:1845590. doi: 10.3389/fspor.2026.1845590 (PMC13341810; doi:10.3389/fspor.2026.1845590)
Supplement: Supplementary file 1 [file Datasheet1.pdf]

zebris Gait Report

Person: [redacted]
Record: [redacted] Gait Analysis FDM-T, Natural arm swing

Pressure plot 3d

Stance, average

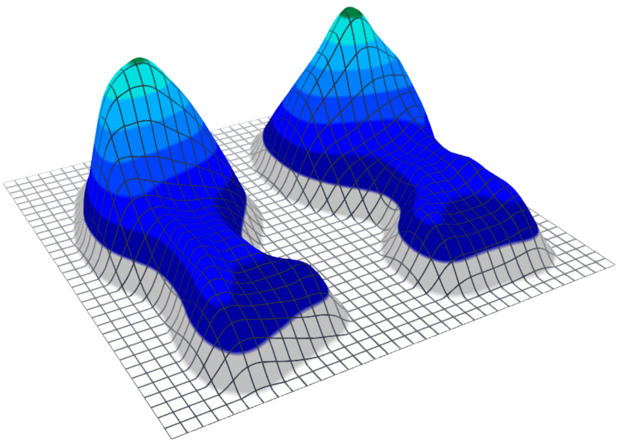

Force forefoot/backfoot

Max load (% of body weight)

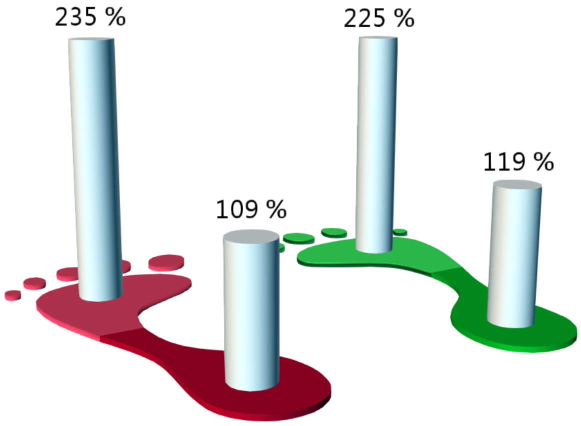

Pressure plots

Stance, average

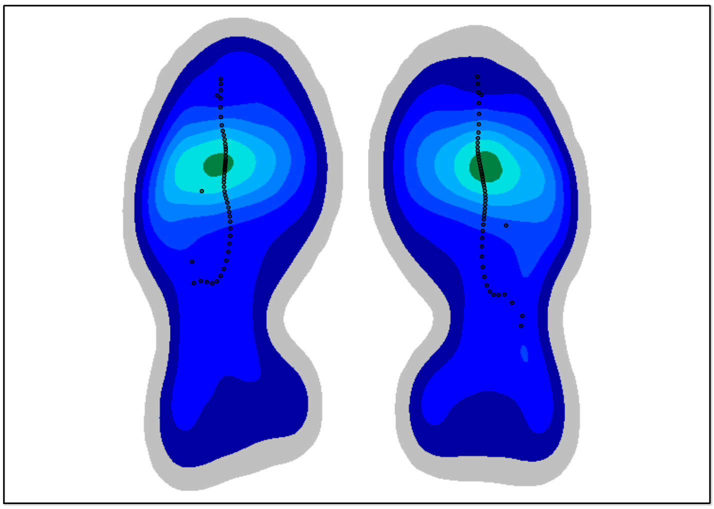

Stance, maximum

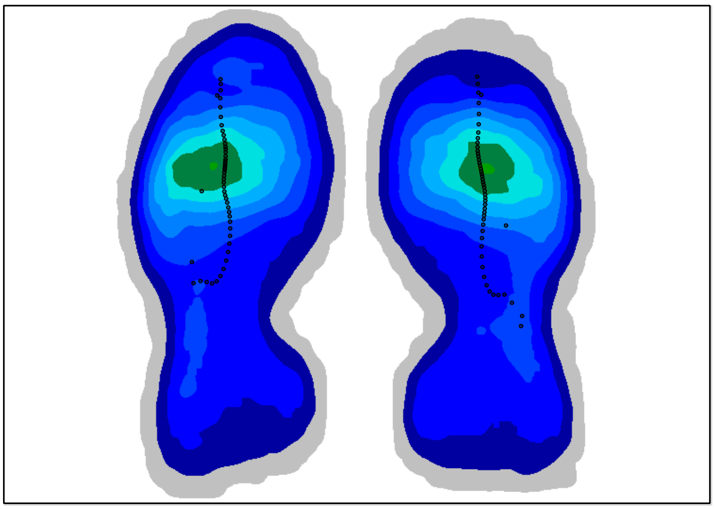

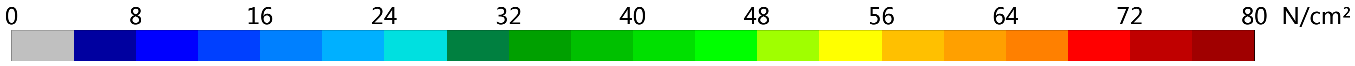

zebris Gait Report

Person: [redacted]
Record: [redacted] Gait Analysis FDM-T, Natural arm swing

Separate footprints

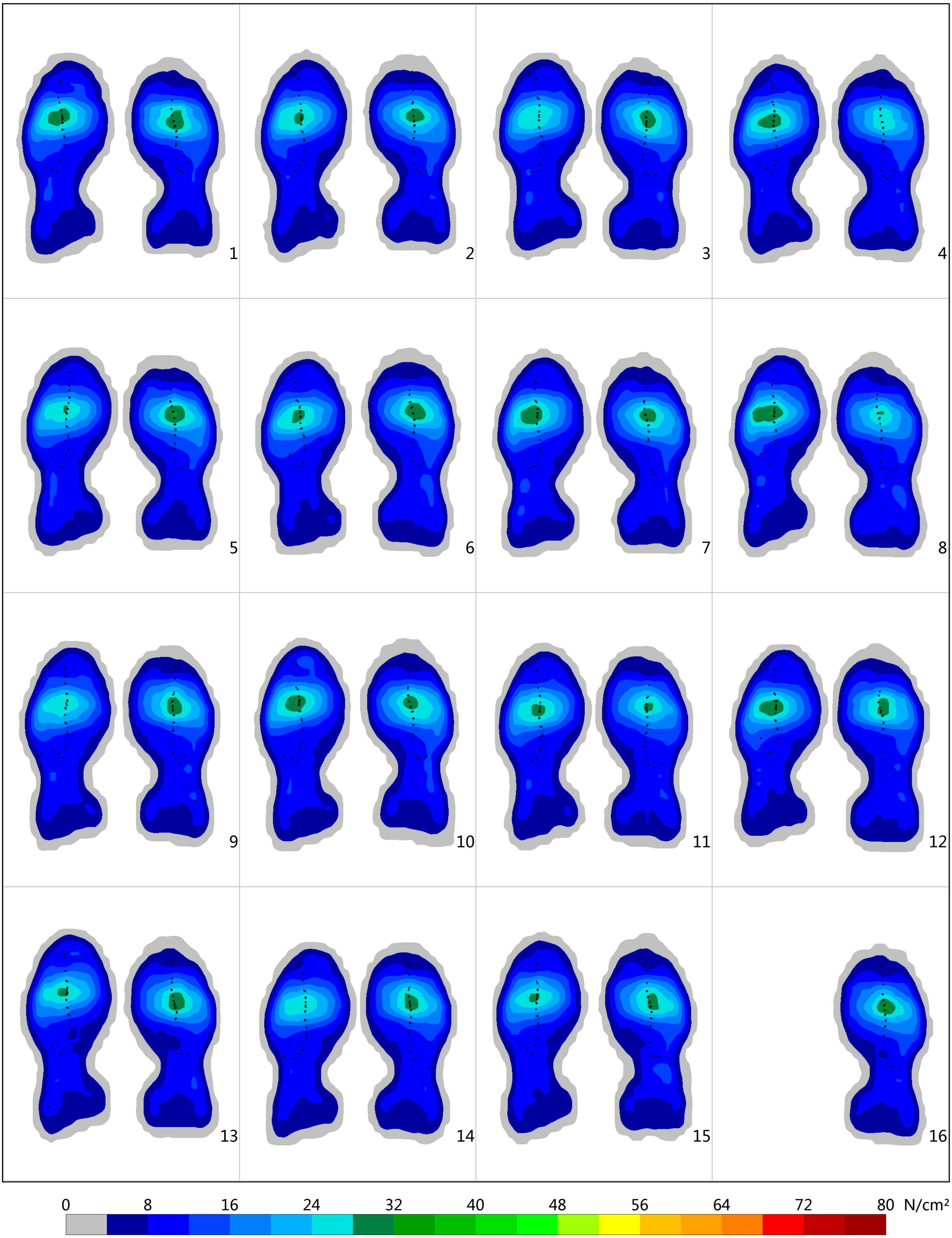

zebris Gait Report

Person: [redacted]
Record: [redacted] Gait Analysis FDM-T, Natural arm swing

zebris Medical GmbH

Gait parameters

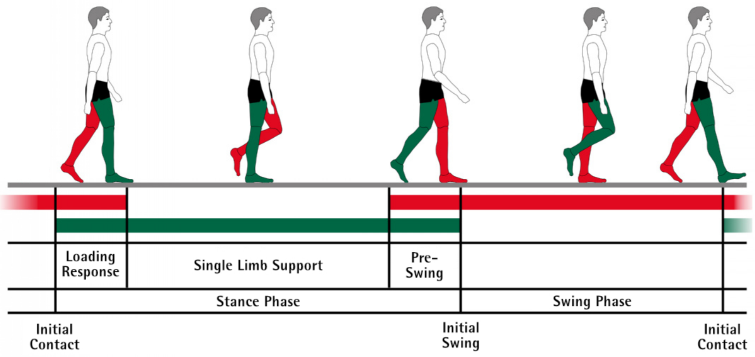

Geometry

Parameter	Side	Value	Scale
Foot rotation, degree	L	-1.2±1.5	-5° … 5°
	R	-3.0±1.4	
Step length, cm	L	111±1	240 cm
	R	111±1	
Stride length, cm		222±1	240 cm
Step width, cm		9±2	

Phases

Parameter	Side	Value	Scale
Stance phase, %	L	30.6±0.7	100 %
	R	30.9±0.7	
Load response, %	L	0.0±0.0	
	R	0.0±0.0	
Single limb support, %	L	30.6±0.7	
	R	31.0±0.6	
Pre-Swing, %	L	0.0±0.0	
	R	0.0±0.0	
Swing phase, %	L	69.4±0.7	
	R	69.1±0.7	
Double stance phase, %		0.0±0.0	

Timing

Parameter	Side	Value	Scale
Step time, sec	L	0.37±0.01	0.8 sec
	R	0.37±0.01	
Stride time, sec		0.74±0.01	0.8 sec
Cadence, steps/min		163±2	180 steps/min
Velocity, km/h		10.8±0.1	12 km/h

zebris Gait Report

Person: [redacted]

Record: [redacted] Gait Analysis FDM-T, Natural arm swing

zebris Medical GmbH

COP analysis

Butterfly

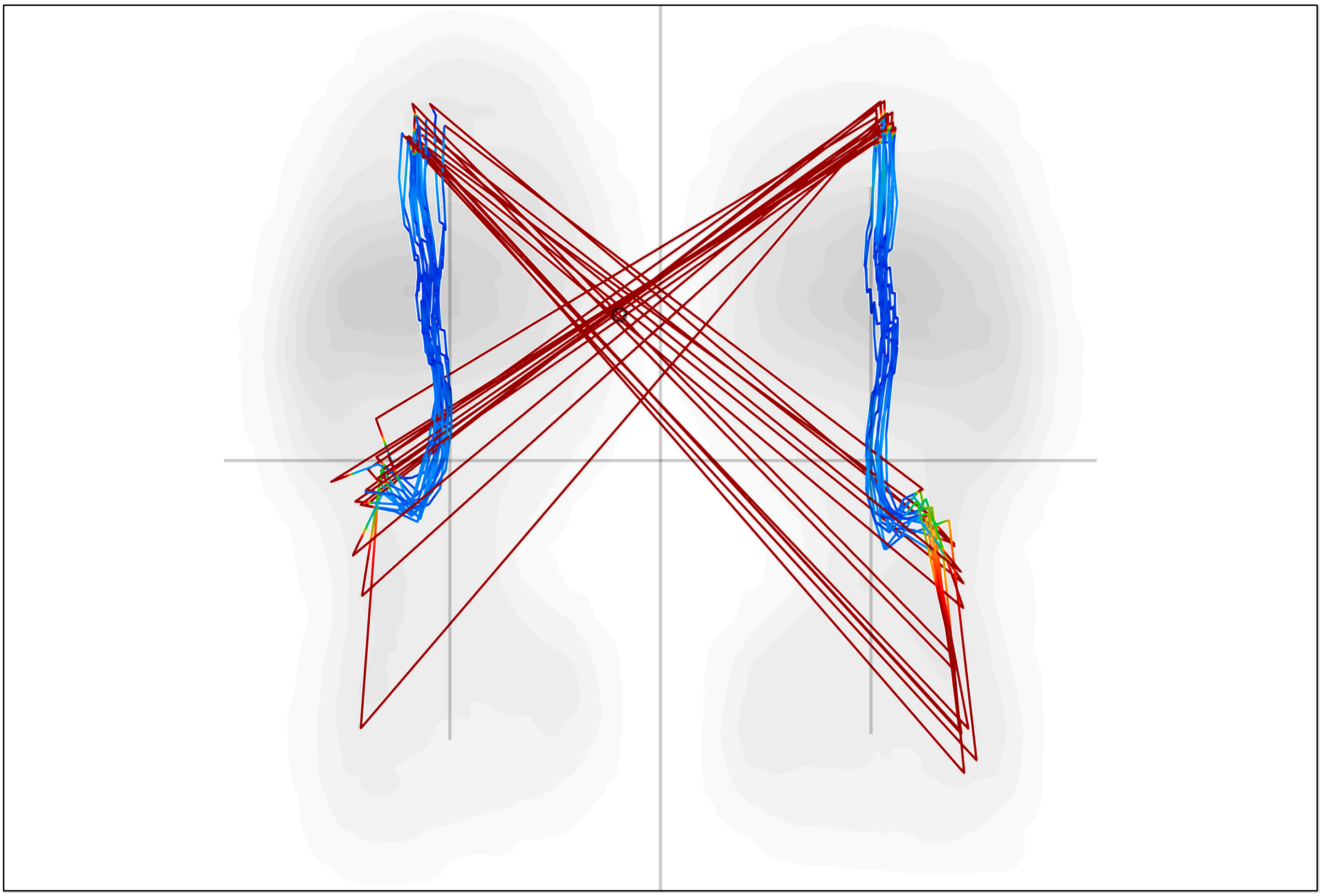

Gait line left	Gait line right
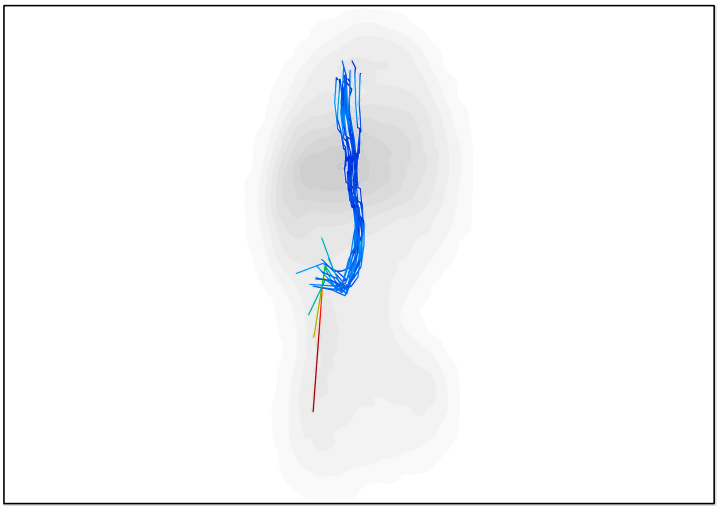	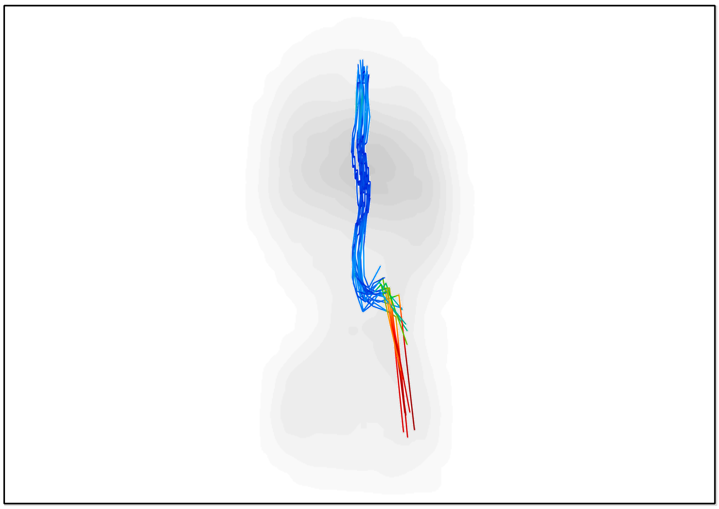

Butterfly parameters

Length of gait line, mm	L	126.7±21.2	210 mm
	R	163.4±29.3	
Single limb support line, mm	L	126.4±22.1	
	R	164.2±28.8	
Ant/post position, mm		46.1±6.5	-105 mm … 105 mm
Lateral symmetry, mm		-13.0±10.4	-25 mm … 25 mm
Max gait line velocity, cm/sec		764.9	

Force and pressure

Pressure curves

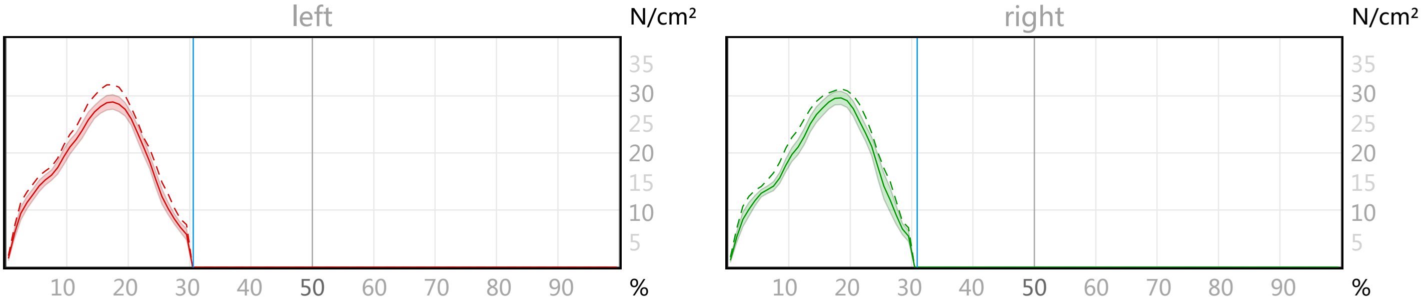

Force curves

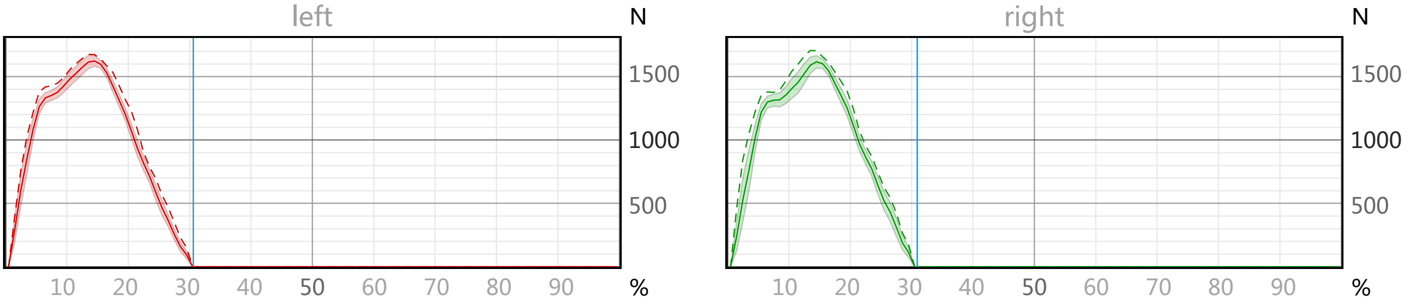

Force parameters

Maximum force1, N	L	1624.0	1800 N
	R	1616.8	
Time maximum force1, %	L	14	100%
	R	14	
Maximum force2, N	L	-	1800 N
	R	-	
Time maximum force2, %	L	-	100%
	R	-	

zebris Gait Report

Person: [redacted]
Record: [redacted] Gait Analysis FDM-T, Natural arm swing

Three foot zone analysis

Zones	Heel Fmax t1	Midfoot (t1+t2)/2	Forefoot Fmax t2	Forefoot Fmax t2	Midfoot (t1+t2)/2	Heel Fmax t1	Zones

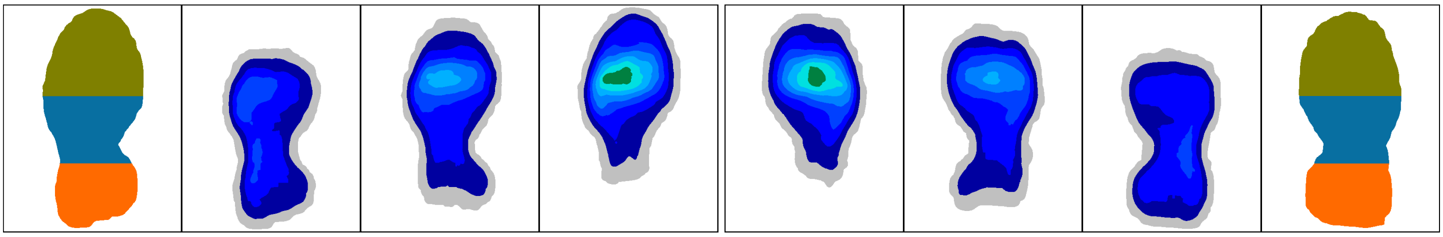

Force overlay

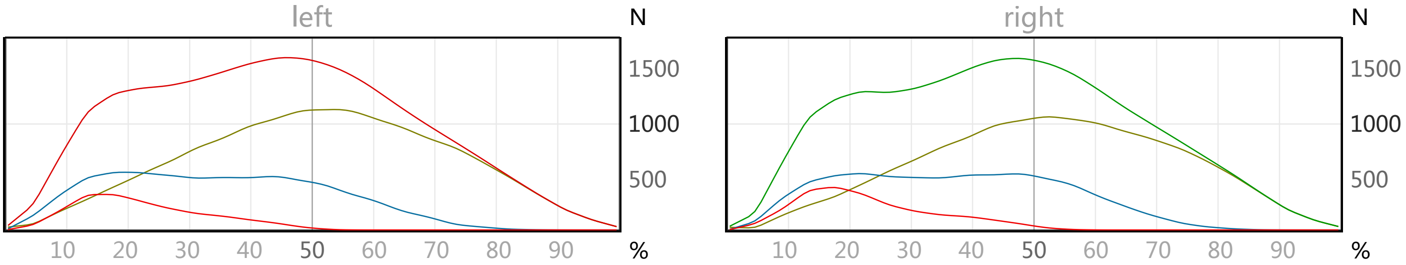

Load change

Time change heel to forefoot, sec	L	0.02±0.01	0.05 sec
	R	0.03±0.01	
Time change heel to forefoot, %	L	7.3±4.3	100%
	R	14.7±4.2	

Maximum force, N

Forefoot	L	1166.3±43.6	1800 N
	R	1095.7±44.5	
Midfoot	L	560.7±28.4	
	R	560.4±30.0	
Heel	L	352.9±25.6	
	R	416.3±34.6	

Maximum pressure, N/cm²

Forefoot	L	29.7±1.4	40 N/cm²
	R	30.8±1.4	
Midfoot	L	20.3±1.8	
	R	20.7±2.0	
Heel	L	12.4±0.8	
	R	12.8±0.8	

Time maximum force, % of stance time

Forefoot	L	47.2±2.9	100%
	R	49.2±2.7	
Midfoot	L	18.0±6.5	
	R	30.9±13.3	
Heel	L	10.3±2.0	
	R	12.0±1.8	

zebris Gait Report

Person: [redacted]

Record: [redacted] Gait Analysis FDM-T, Natural arm swing

zebris Medical GmbH

Contact time, % of stance time

Forefoot	L	95.0±1.4
	R	93.2±2.1
Midfoot	L	80.9±2.9
	R	79.8±2.7
Heel	L	48.4±2.2
	R	50.8±2.6

100%

Seven foot zone analysis

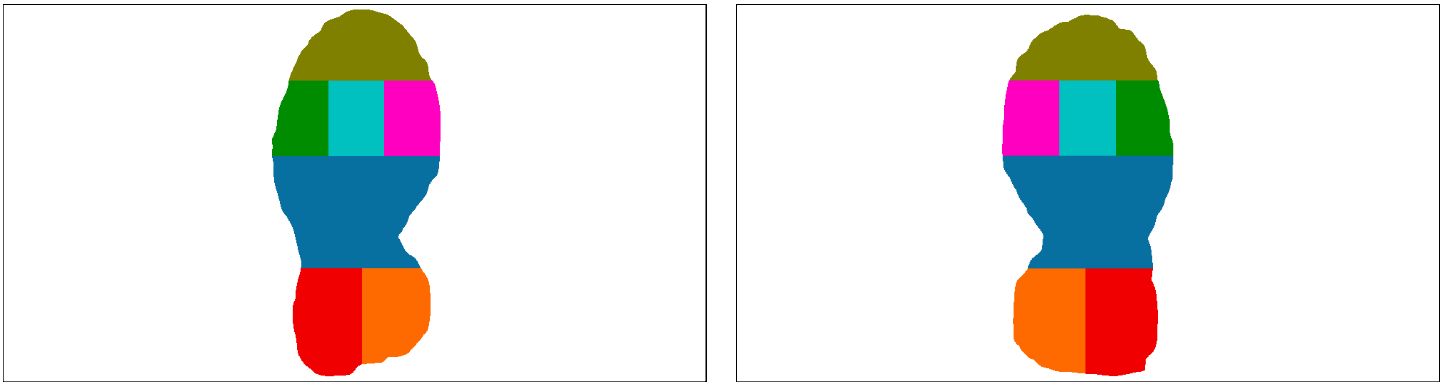

Average Force

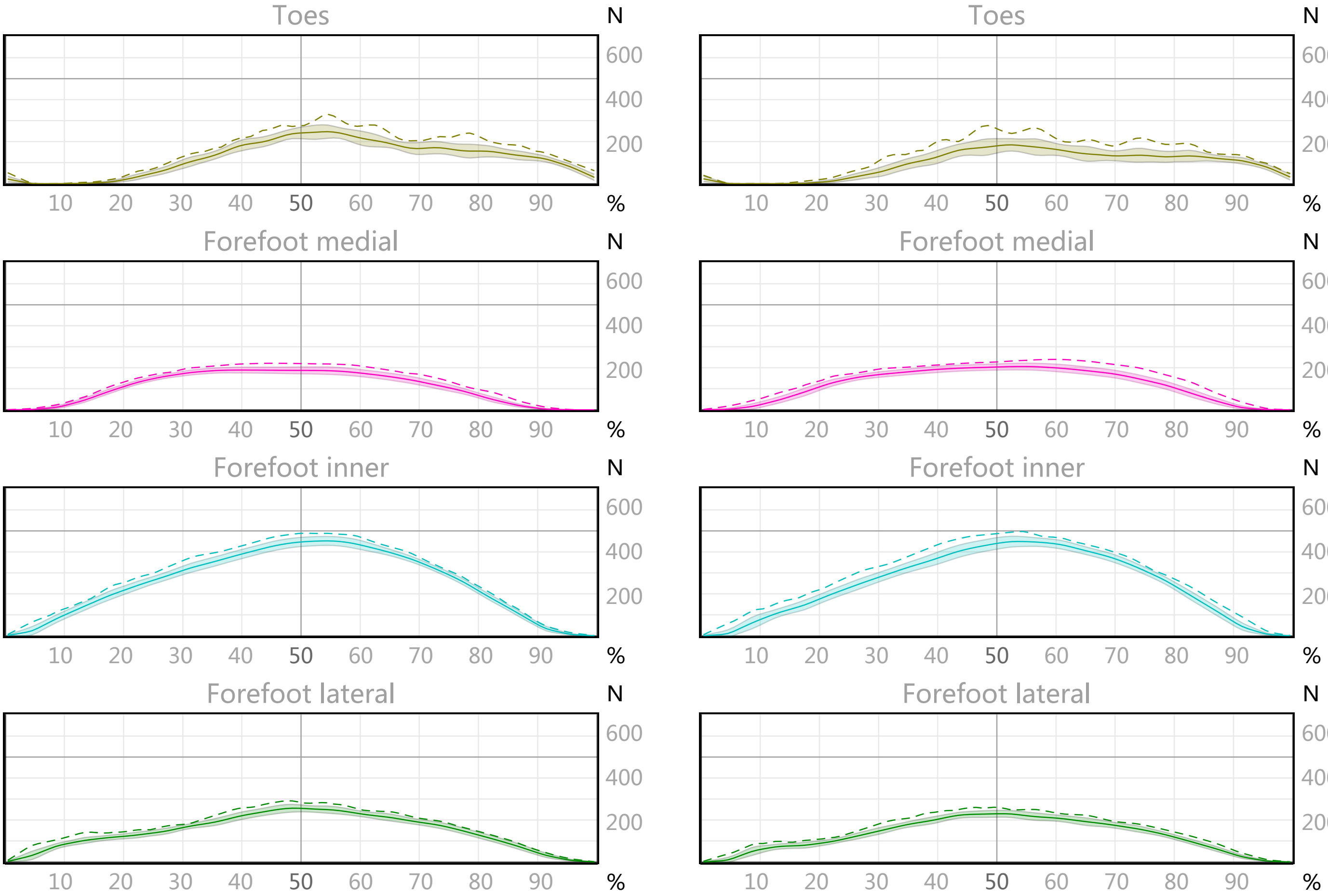

zebris Gait Report

Person: [redacted]
Record: [redacted] Gait Analysis FDM-T, Natural arm swing

zebris Medical GmbH

Average Force

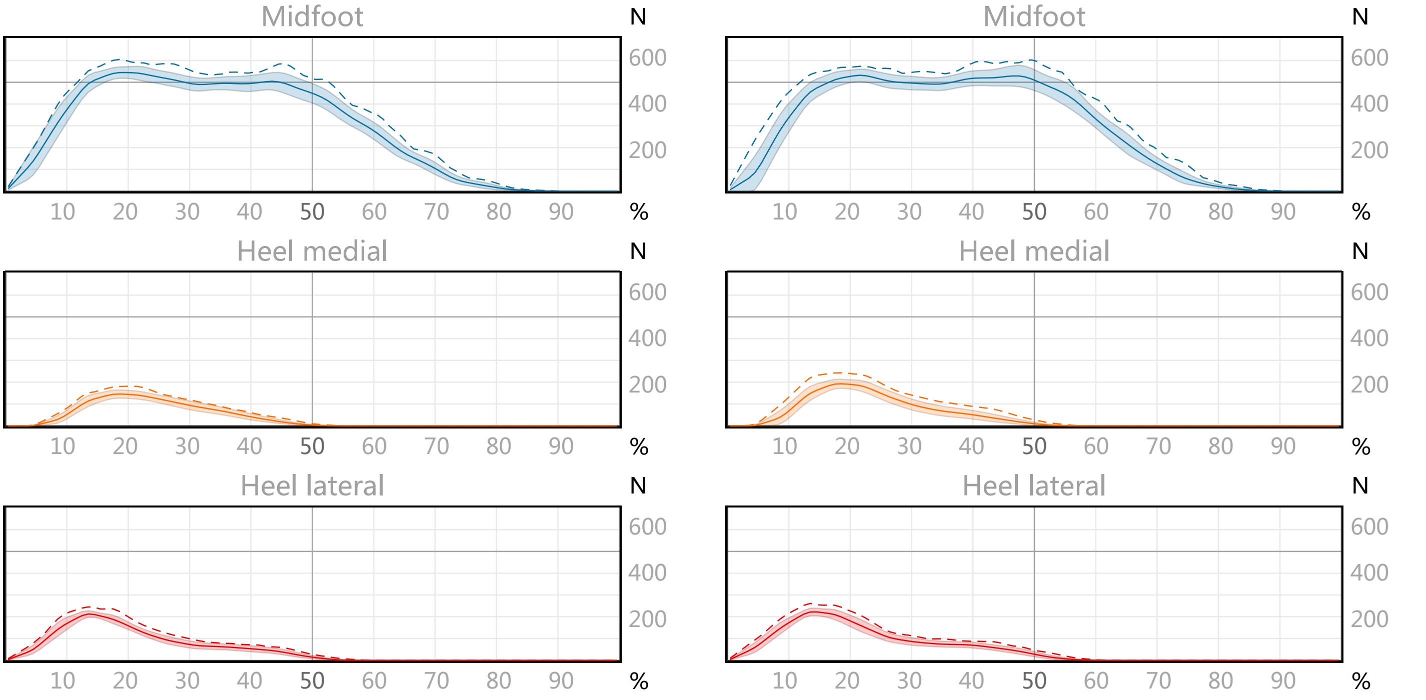

Average Max Pressure

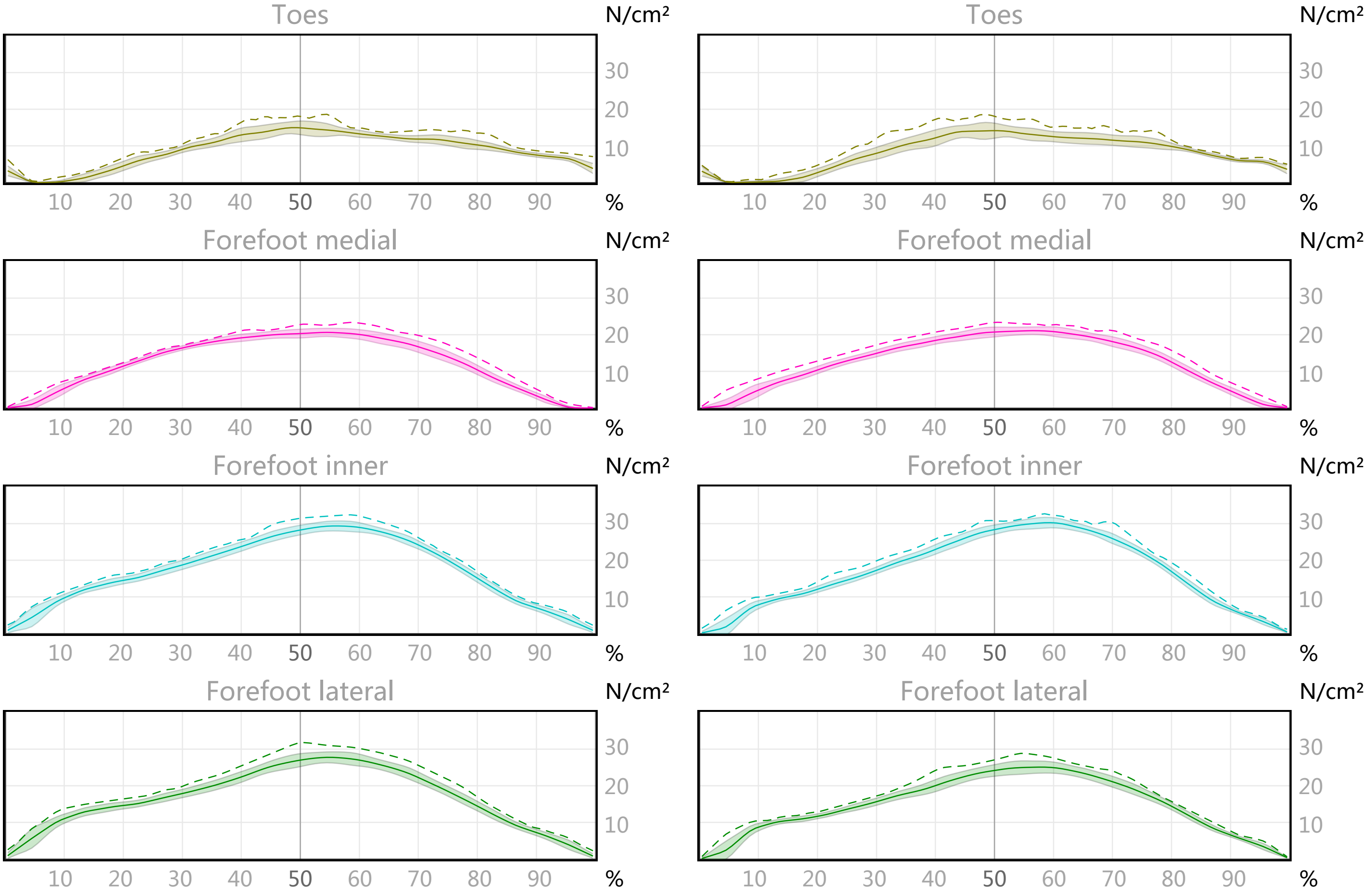

zebris Gait Report

Person: [redacted]
Record: [redacted] Gait Analysis FDM-T, Natural arm swing

Average Max Pressure

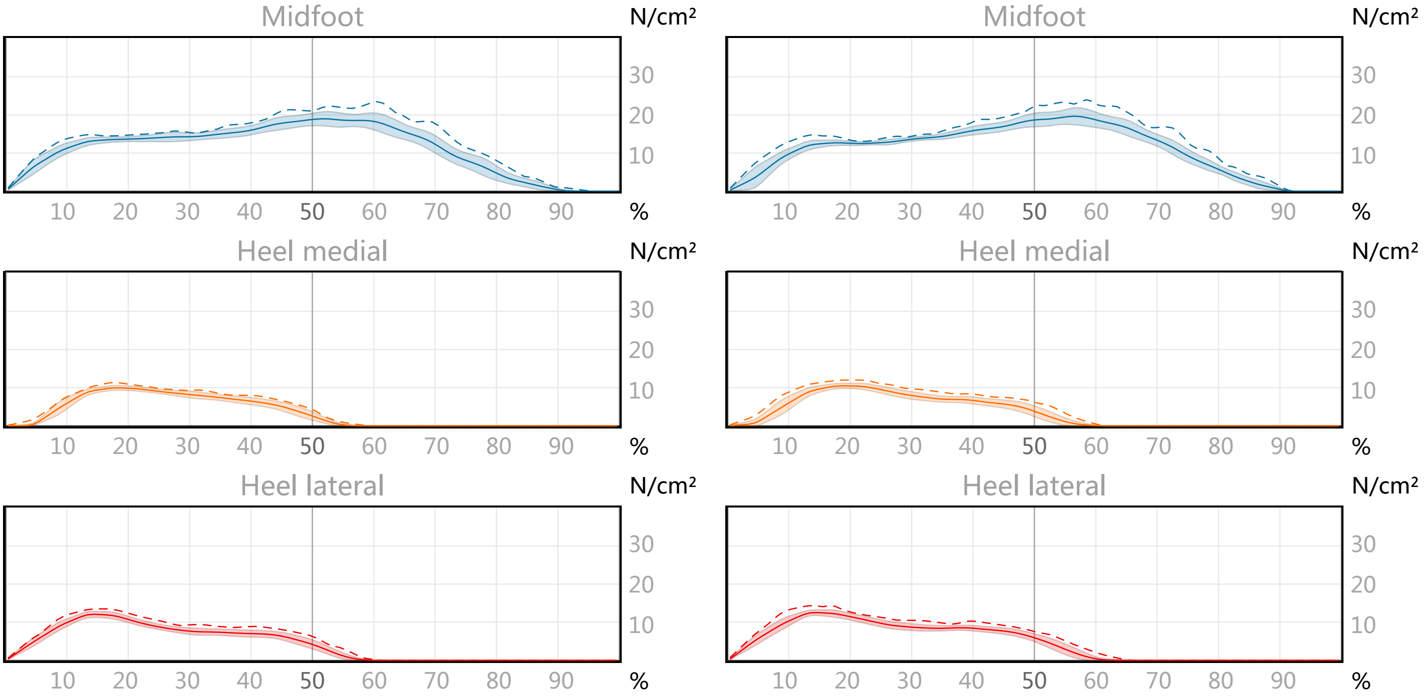

Contact time, % of stance time

Toes	L	85.0±2.5	100%
	R	82.8±4.5	
Forefoot medial	L	83.8±2.4	
	R	85.0±3.2	
Forefoot inner	L	92.1±3.1	
	R	88.6±2.5	
Forefoot lateral	L	92.4±2.8	
	R	88.8±2.1	
Midfoot	L	80.9±2.9	
	R	79.8±2.7	
Heel medial	L	41.6±2.6	
	R	45.1±3.1	
Heel lateral	L	48.4±2.2	
	R	50.8±2.6	

Maximum force, N

Toes	L	268.0±31.4	700 N
	R	210.5±34.1	
Forefoot medial	L	193.2±15.1	
	R	207.7±17.1	
Forefoot inner	L	454.5±20.7	
	R	453.8±23.5	
Forefoot lateral	L	262.7±18.1	
	R	238.8±14.9	
Midfoot	L	560.7±28.4	
	R	560.4±30.0	
Heel medial	L	149.4±19.5	
	R	200.4±18.9	
Heel lateral	L	223.0±14.2	
	R	230.9±18.4	

zebris Gait Report

Person: [redacted]
Record: [redacted] Gait Analysis FDM-T, Natural arm swing

Time maximum force, % of stance time

Region	Side	Value (100%)
Toes	L	48.4±3.4
	R	47.3±7.1
Forefoot medial	L	37.7±7.3
	R	50.0±3.5
Forefoot inner	L	49.8±2.5
	R	50.0±3.2
Forefoot lateral	L	44.9±2.9
	R	45.1±3.3
Midfoot	L	18.0±6.5
	R	30.9±13.3
Heel medial	L	15.0±2.6
	R	14.5±2.5
Heel lateral	L	9.4±1.4
	R	10.1±2.0
