# Supplementary material for: Aristotle's arm-swing hypothesis: biomechanical evidence from forward and inverse dynamics in an Olympic sprinter
Source: Front Sports Act Living. 2026 Jun 24;8:1845590. doi: 10.3389/fspor.2026.1845590 (PMC13341810; doi:10.3389/fspor.2026.1845590)
Supplement: Supplementary file 2 [file Datasheet2.pdf]

zebris Gait Report

Person: [redacted]
Record: [redacted] Gait Analysis FDM-T, Modified arm swing

Pressure plot 3d

Stance, average

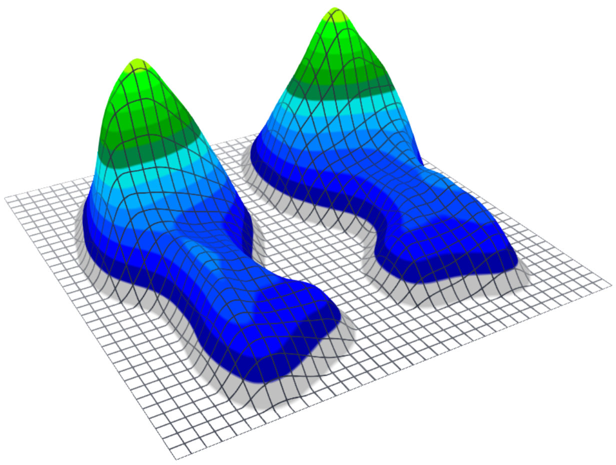

Force forefoot/backfoot

Max load (% of body weight)

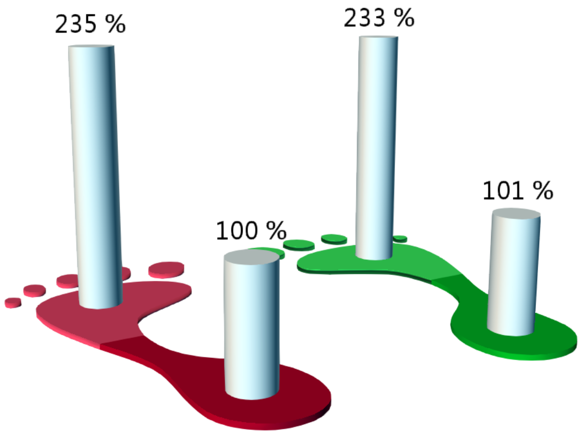

Pressure plots

Stance, average

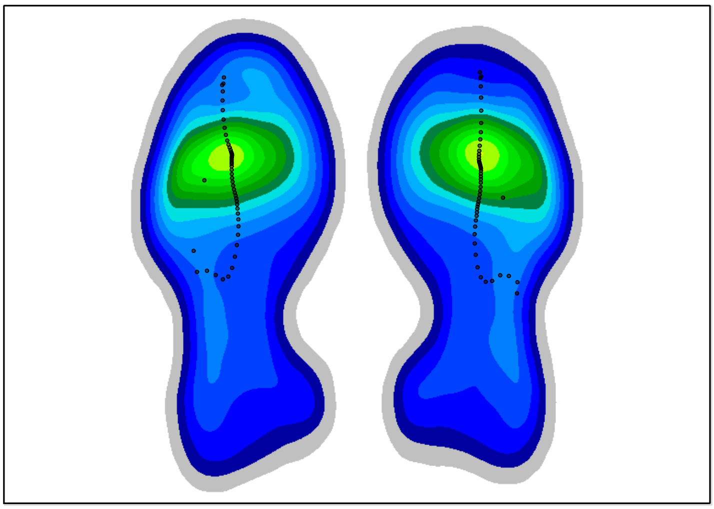

Stance, maximum

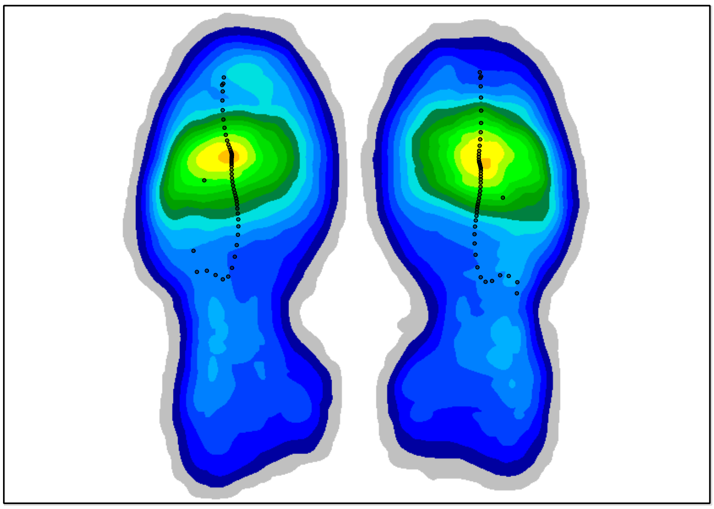

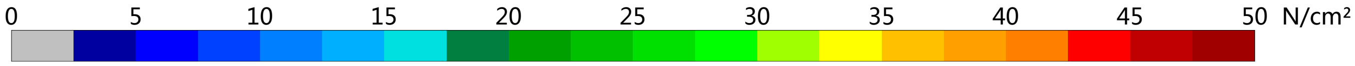

zebris Gait Report

Person: [redacted]
Record: [redacted] Gait Analysis FDM-T, Modified arm swing

Separate footprints

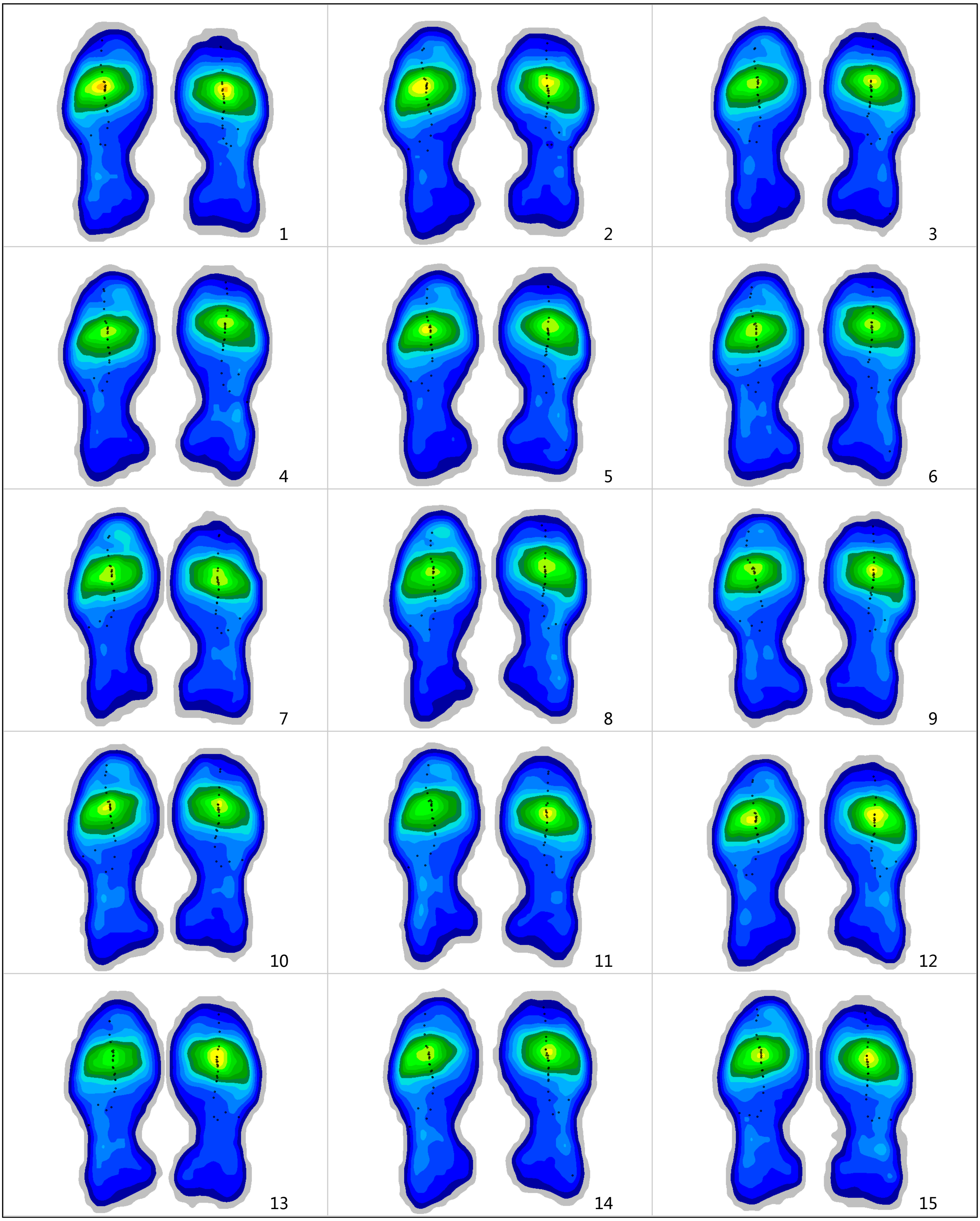

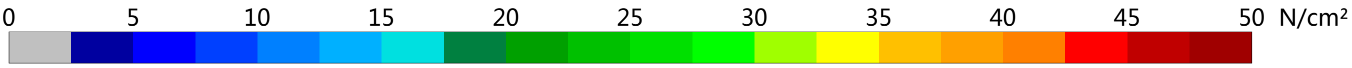

zebris Gait Report

Person: [redacted]
Record: [redacted] Gait Analysis FDM-T, Modified arm swing

zebris Medical GmbH

Gait parameters

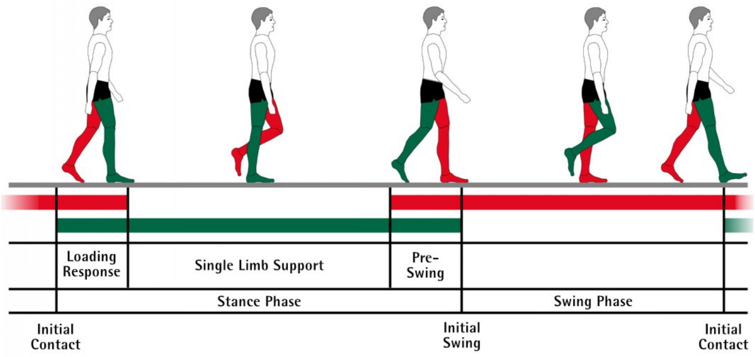

Geometry

Parameter	Side	Value	Scale
Foot rotation, degree	L	1.9±1.3	-4° … 4°
	R	1.1±1.7	
Step length, cm	L	116±2	250 cm
	R	114±2	
Stride length, cm		230±3	250 cm
Step width, cm		7±2	

Phases

Parameter	Side	Value	Scale
Stance phase, %	L	30.5±0.8	100 %
	R	31.3±1.0	
Load response, %	L	0.0±0.0	
	R	0.0±0.0	
Single limb support, %	L	30.5±0.8	
	R	31.4±1.0	
Pre-Swing, %	L	0.0±0.0	
	R	0.0±0.0	
Swing phase, %	L	69.5±0.8	
	R	68.7±1.0	
Double stance phase, %		0.0±0.0	

Timing

Parameter	Side	Value	Scale
Step time, sec	L	0.39±0.01	0.9 sec
	R	0.38±0.01	
Stride time, sec		0.77±0.01	0.9 sec
Cadence, steps/min		156±2	170 steps/min
Velocity, km/h		10.8±0.1	12 km/h

COP analysis

Butterfly

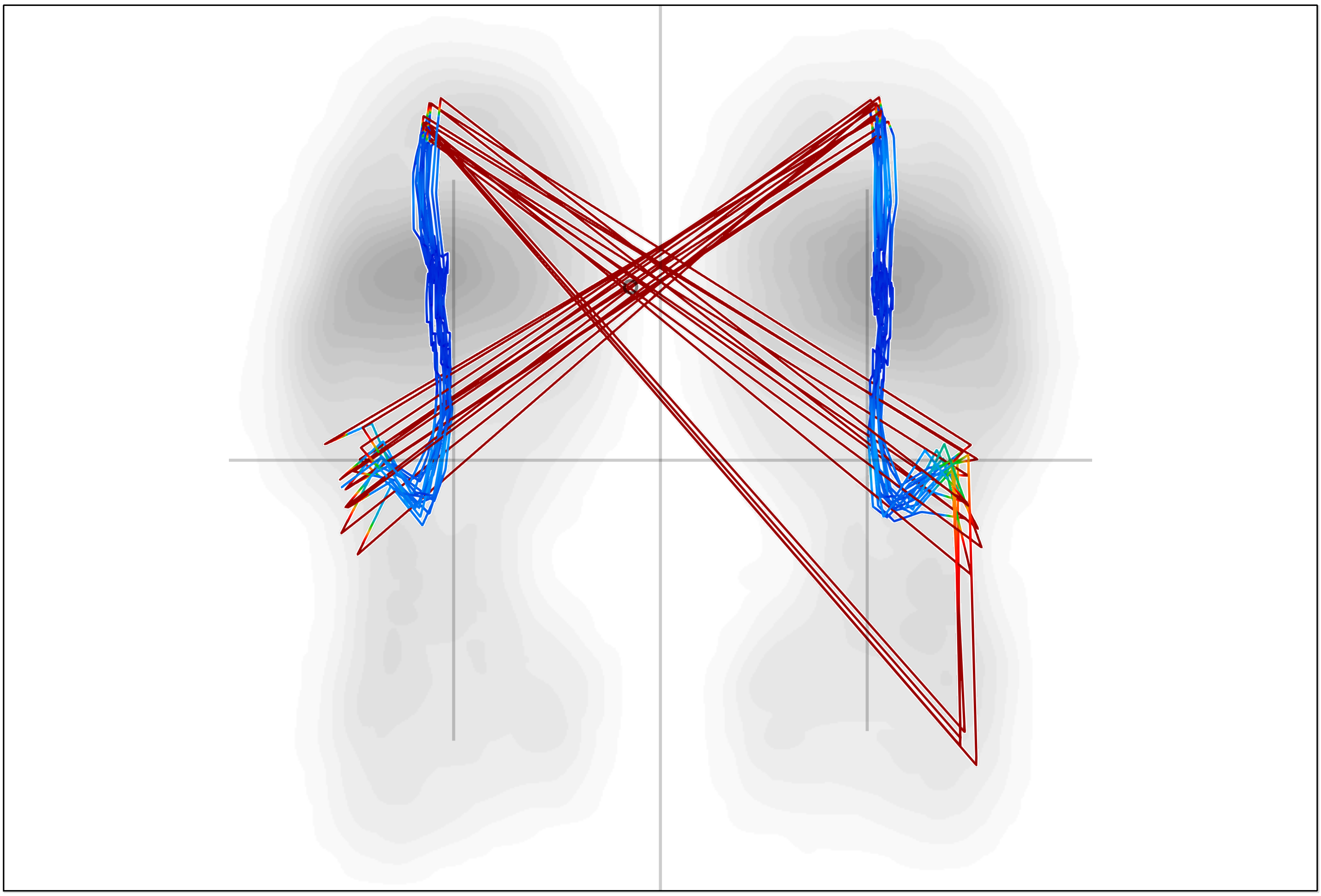

Gait line left

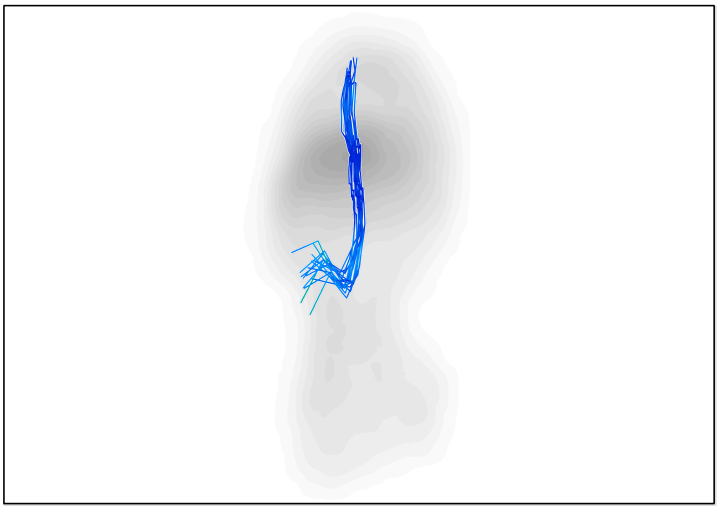

Gait line right

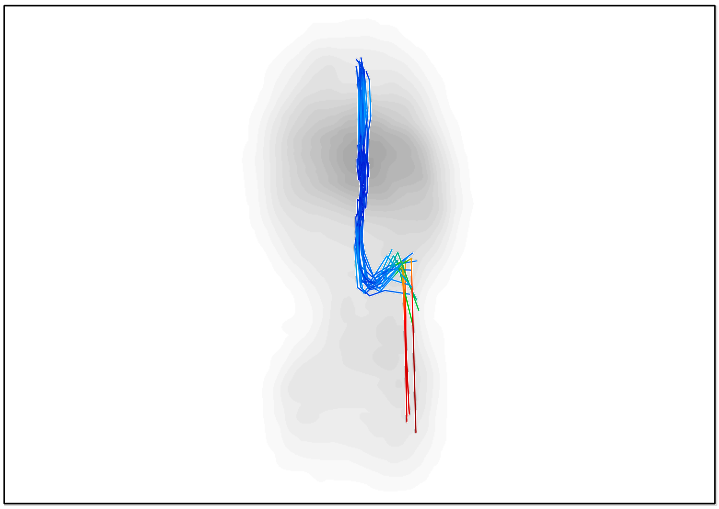

Butterfly parameters

Length of gait line, mm	L	125.3±6.7	210 mm
	R	151.0±35.1	
Single limb support line, mm	L	124.6±6.6	
	R	154.3±36.2	
Ant/post position, mm		56.4±7.5	-105 mm / 105 mm
Lateral symmetry, mm		-9.7±10.2	-21 mm / 21 mm
Max gait line velocity, cm/sec		1008.3	

zebris Gait Report

Person: [redacted]
Record: [redacted] Gait Analysis FDM-T, Modified arm swing

Force and pressure

Pressure curves

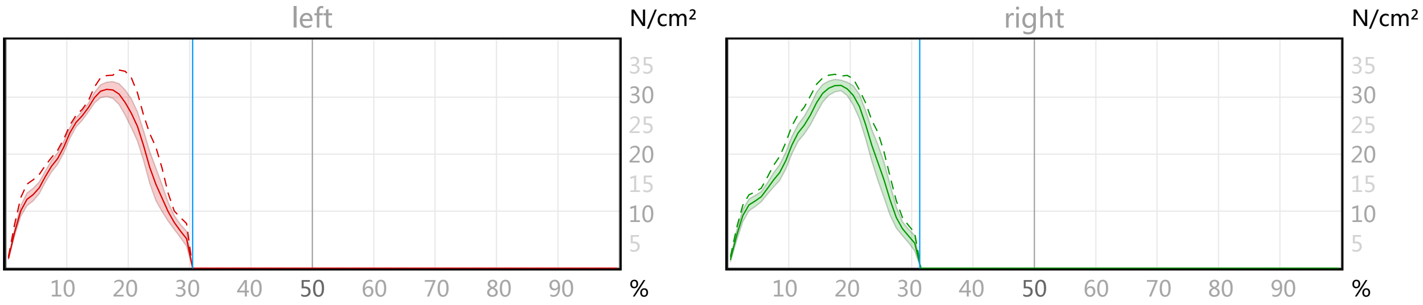

Force curves

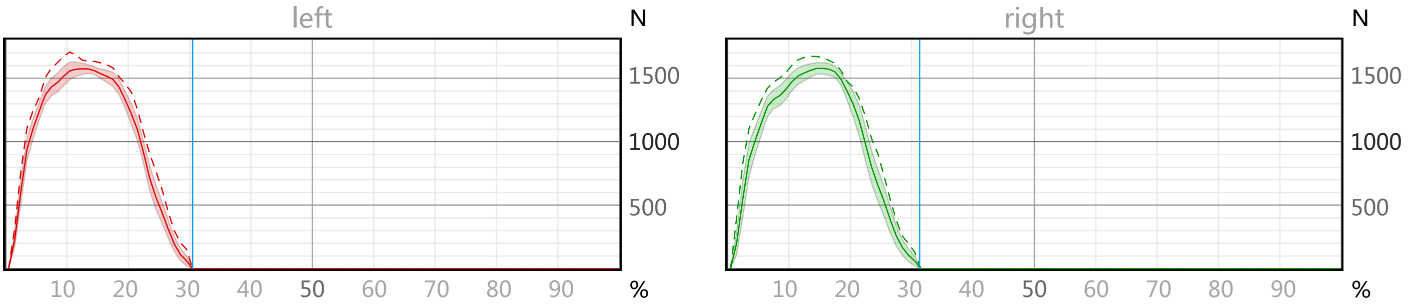

Force parameters

Maximum force1, N	L	1574.5	1800 N
	R	1578.4	
Time maximum force1, %	L	12	100%
	R	15	
Maximum force2, N	L	-	1800 N
	R	-	
Time maximum force2, %	L	-	100%
	R	-	

zebris Gait Report

Person: [redacted]
Record: [redacted] Gait Analysis FDM-T, Modified arm swing

Three foot zone analysis

Zones	Heel Fmax t1	Midfoot (t1+t2)/2	Forefoot Fmax t2	Forefoot Fmax t2	Midfoot (t1+t2)/2	Heel Fmax t1	Zones

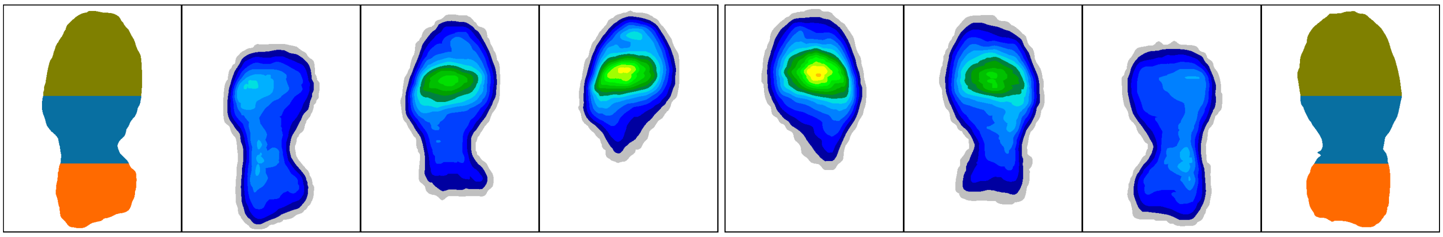

Force overlay

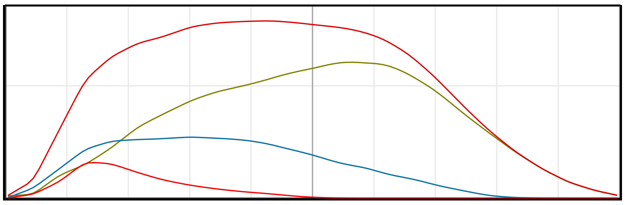

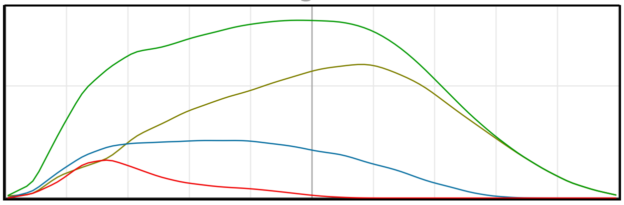

Load change

Time change heel to forefoot, sec	L	0.01±0.00	0.017 sec
	R	0.01±0.01	
Time change heel to forefoot, %	L	2.7±2.1	100%
	R	3.2±3.2	

Maximum force, N

Forefoot	L	1233.2±48.5	1700 N
	R	1214.6±42.5	
Midfoot	L	561.7±33.1	
	R	533.8±39.7	
Heel	L	332.6±46.6	
	R	347.0±27.0	

Maximum pressure, N/cm²

Forefoot	L	32.7±1.8	40 N/cm²
	R	33.4±1.0	
Midfoot	L	19.0±1.7	
	R	18.6±1.9	
Heel	L	11.6±1.4	
	R	12.1±1.3	

Time maximum force, % of stance time

Forefoot	L	52.3±3.1	100%
	R	55.0±2.0	
Midfoot	L	27.0±3.1	
	R	30.3±5.3	
Heel	L	9.9±1.9	
	R	11.4±1.8	

zebris Gait Report

Person: [redacted]
Record: [redacted] Gait Analysis FDM-T, Modified arm swing

Contact time, % of stance time

Forefoot	L	95.7±0.1	100%
	R	94.8±1.7	
Midfoot	L	78.7±2.3	
	R	77.3±3.2	
Heel	L	45.8±2.0	
	R	50.8±1.7	

Seven foot zone analysis

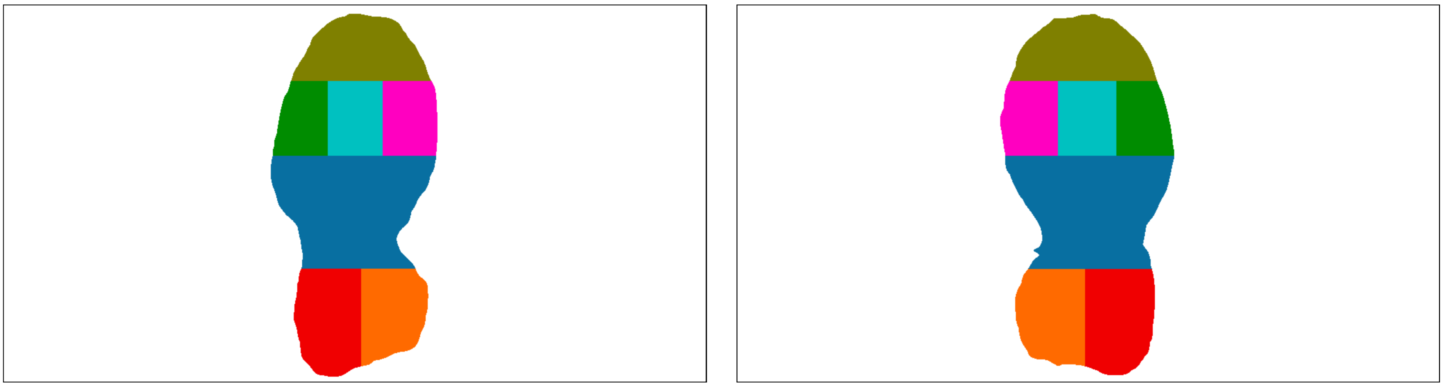

Average Force

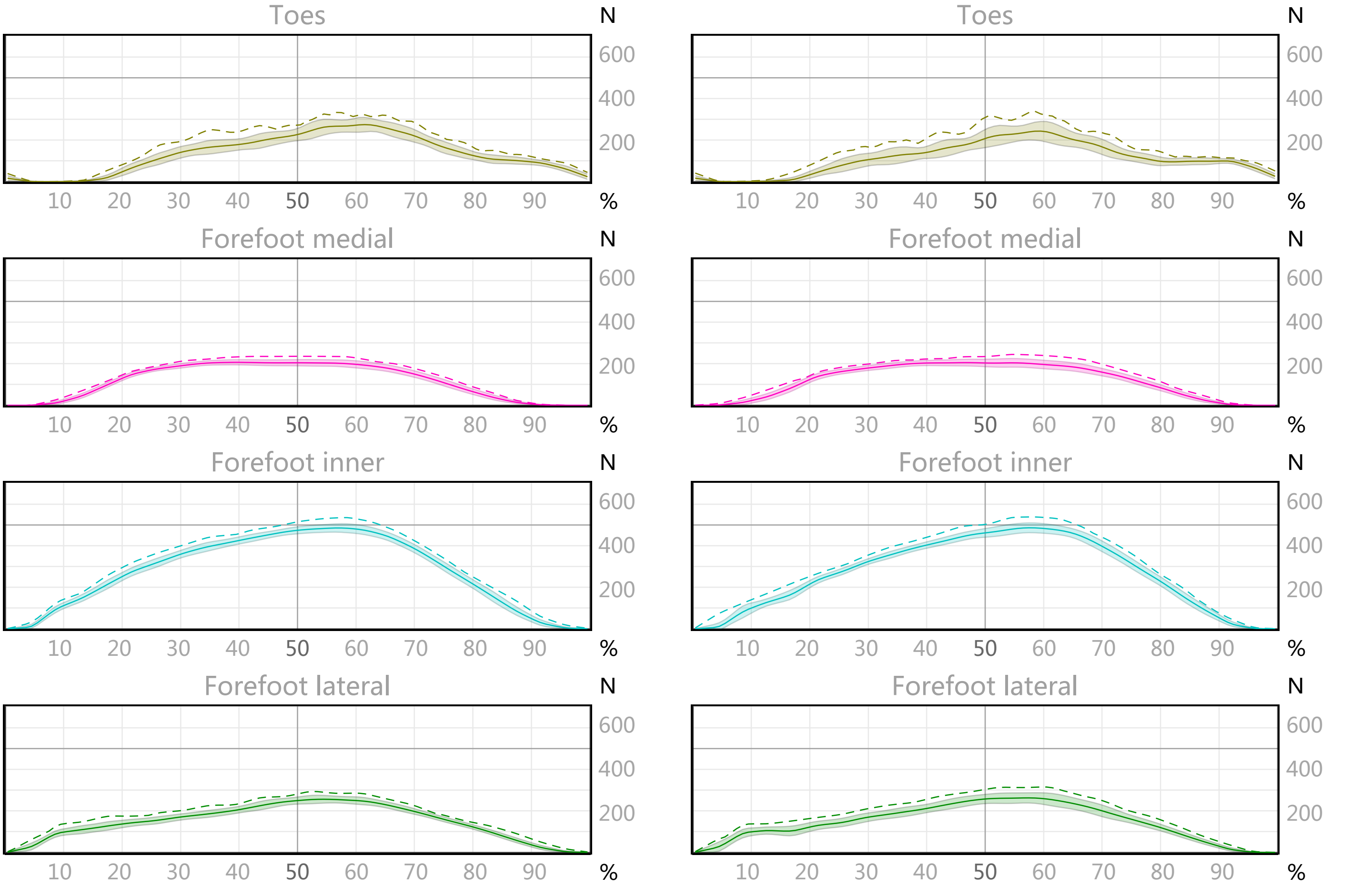

zebris Gait Report

Person: [redacted]

Record: [redacted] Gait Analysis FDM-T, Modified arm swing

Average Force

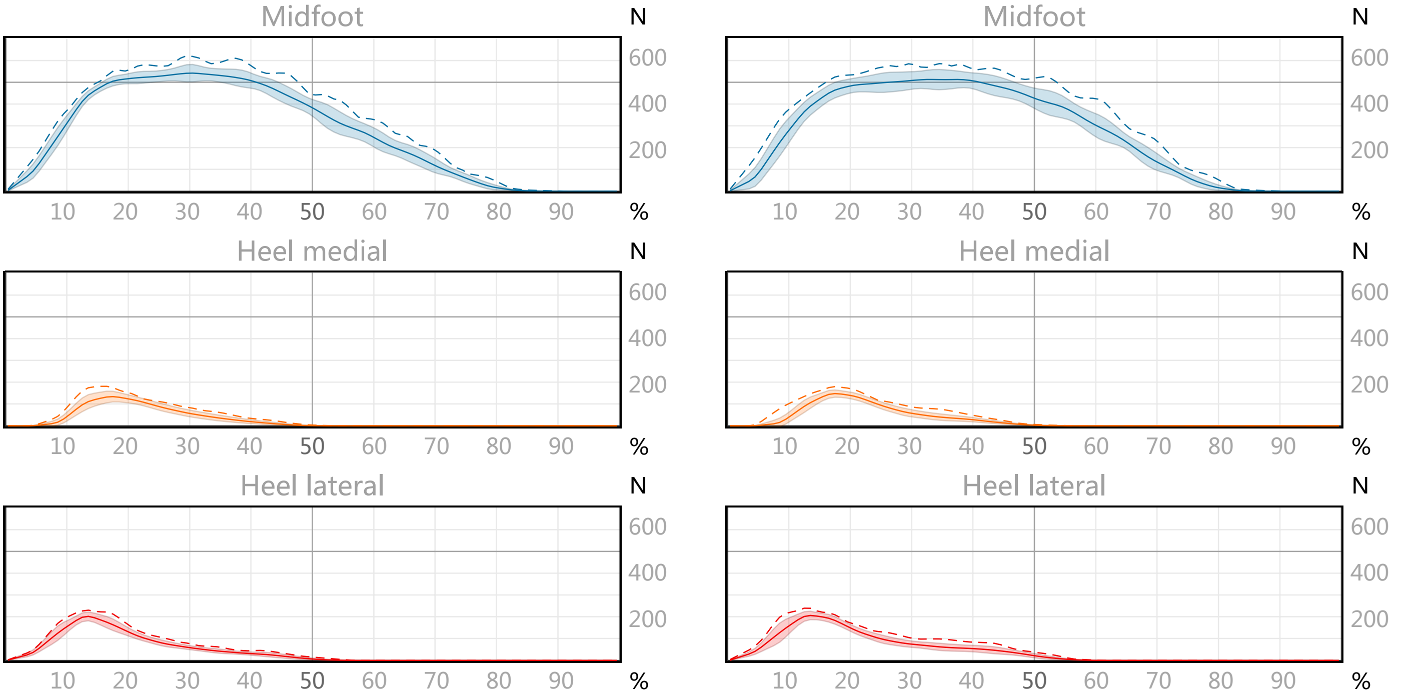

Average Max Pressure

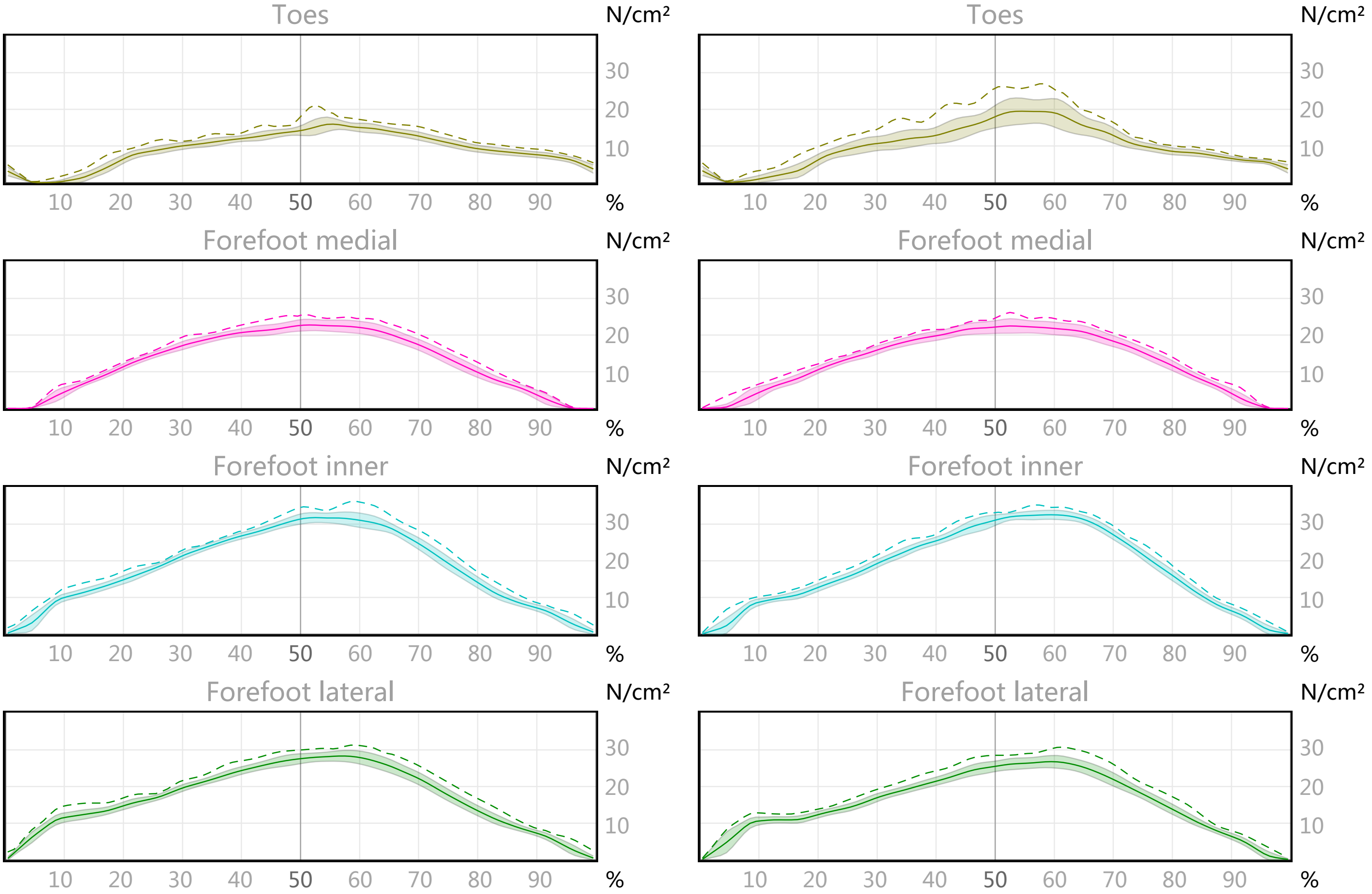

zebris Gait Report

Person: [redacted]
Record: [redacted] Gait Analysis FDM-T, Modified arm swing

Average Max Pressure

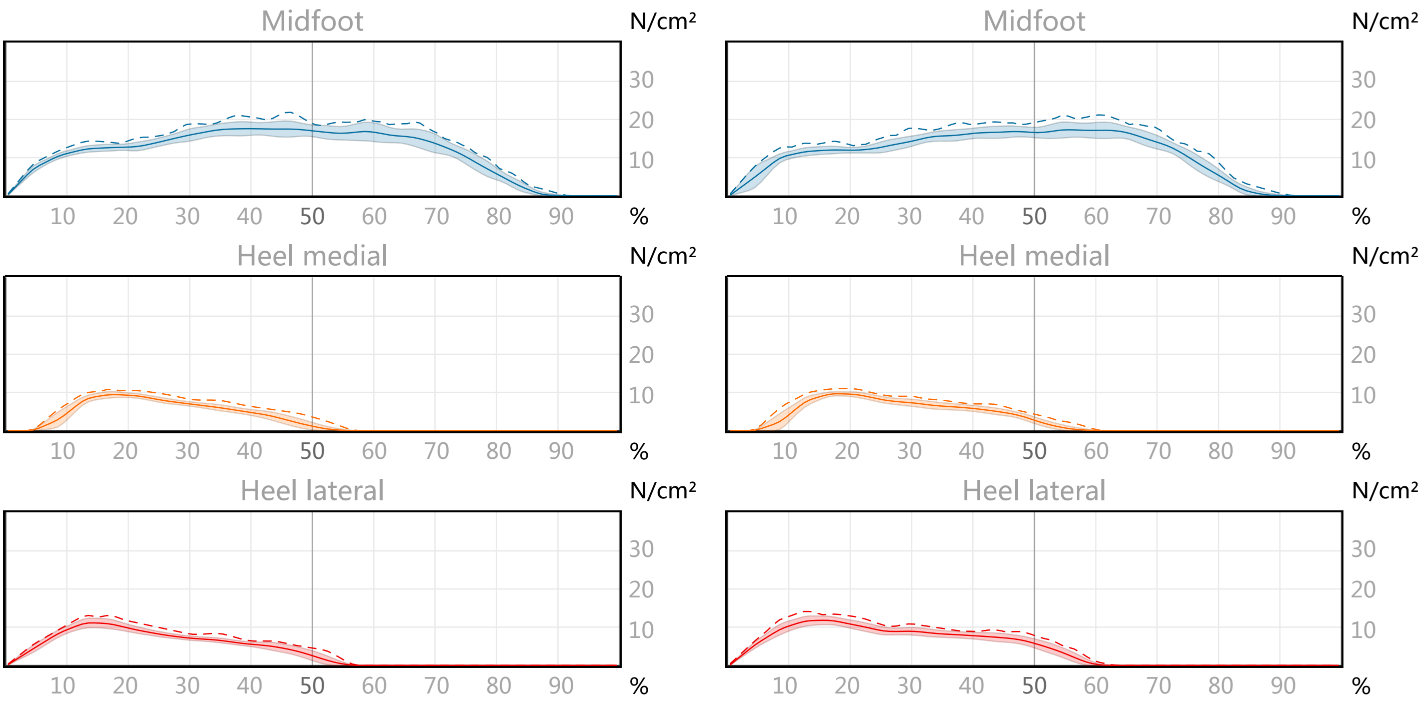

Contact time, % of stance time

			100%
Toes	L	85.5±2.6	
	R	88.3±2.7	
Forefoot medial	L	81.5±1.9	
	R	81.7±2.2	
Forefoot inner	L	90.4±2.4	
	R	87.6±3.2	
Forefoot lateral	L	91.2±1.8	
	R	88.1±2.7	
Midfoot	L	78.7±2.3	
	R	77.3±3.2	
Heel medial	L	38.4±3.6	
	R	42.3±3.4	
Heel lateral	L	45.8±2.0	
	R	50.8±1.7	

Maximum force, N

			700 N
Toes	L	295.5±32.1	
	R	268.4±43.2	
Forefoot medial	L	210.6±12.8	
	R	210.2±17.4	
Forefoot inner	L	487.8±20.7	
	R	489.4±23.7	
Forefoot lateral	L	260.5±18.5	
	R	267.1±24.6	
Midfoot	L	561.7±33.1	
	R	533.8±39.7	
Heel medial	L	139.3±24.5	
	R	153.1±16.9	
Heel lateral	L	210.5±20.9	
	R	214.8±19.8	

Time maximum force, % of stance time

Region	Side	Value
Toes	L	55.1±3.7
	R	54.2±3.6
Forefoot medial	L	41.7±7.4
	R	41.6±6.7
Forefoot inner	L	52.2±2.0
	R	53.9±1.8
Forefoot lateral	L	50.3±2.0
	R	52.3±4.4
Midfoot	L	27.0±3.1
	R	30.3±5.3
Heel medial	L	13.1±2.0
	R	13.6±1.9
Heel lateral	L	8.8±1.0
	R	9.4±1.6

100%
