# Supplementary material for: Aristotle's arm-swing hypothesis: biomechanical evidence from forward and inverse dynamics in an Olympic sprinter
Source: Front Sports Act Living. 2026 Jun 24;8:1845590. doi: 10.3389/fspor.2026.1845590 (PMC13341810; doi:10.3389/fspor.2026.1845590)
Supplement: Supplementary file 3 [file Datasheet3.pdf]

Aristotle's Arm-Swing Hypothesis: Biomechanical Evidence from Forward and Inverse Dynamics in an Olympic Sprinter

Supplementary Materials

This file includes:

Supplementary Information

References

Figures S1-S9

Table S1

Other Supplementary Materials for this manuscript include the following:

Data files (Test Report S1 and S2)

1 APPENDIX A

1.1 The Lagrangian Equations for the Upper and Lower Limbs

The advent of dynamical systems theory has prompted humanity to re-examine the world: nature is an ordered, bounded, periodic-orbit system with sensitive dependence on initial conditions, and is essentially a chaotic complex system (Prigogine and Stengers, 2018). The human body, as a complex system (Juarrero, 2000), exhibits self-organization at the microscopic level (Camazine et al., 2020) and self-similarity at the macroscopic level (Aristidou et al., 2018). Self-similarity implies that, as the resolution (grid size) increases, An object is considered fractal when its measured volume varies with the measurement scale across multiple orders of magnitude and follows a power-law scaling with a non-integer exponent. In this sense, the proteins and molecules of every individual are structurally similar, rendering the human form fractal.

Furthermore, the structure of the human limbs—the upper limb (hand, forearm, and upper arm) and the lower limb (foot, shank, and thigh)—can be modeled as a triple pendulum (Agarana and Akinlabi, 2018). The motion of a triple pendulum is governed by a set of coupled ordinary differential equations, and is inherently chaotic (Li and Yorke, 1975). Based on Lagrangian mechanics (Brizard, 2014), the Lagrangian equations for the upper and lower limbs can be formulated separately to evaluate the mechanical efficiency of limb swing.

1.2 Lagrangian Equation of the Upper Limbs

The bilateral arm swing is simplified into two triple-pendulum structures, each consisting of the hand, forearm, and upper arm. The Lagrangian for each segment of the upper limbs is formulated to describe their swing motion. Based on Lagrangian mechanics (Brizard, 2014), the Lagrangian equations of motion for the upper limbs are established. Using these equations, the Lagrangians of the left and right arms are computed. Combined with motion capture data and existing segmental inertial parameters of the human body (De Leva, 1996), the mechanical efficiency of arm swing during running is evaluated.

The formulation of the Lagrangians for each segment of the upper limbs is as follows:

Let the coordinates of the centers of mass (COM) of the left upper arm, left forearm, and left hand be (x_1^l, y_1^l, z_1^l), (x_2^l, y_2^l, z_2^l), and (x_3^l, y_3^l, z_3^l), respectively; and those of the right upper arm, right forearm, and right hand be (x_1^r, y_1^r, z_1^r), (x_2^r, y_2^r, z_2^r) and (x_3^r, y_3^r, z_3^r), respectively. Let the lengths of the left upper arm, left forearm, and left hand be l_1^l, l_2^l, and l_3^l, respectively; and those of the right upper arm, right forearm, and right hand be l_1^r, l_2^r, and l_3^r, respectively. The angles between the left upper arm and trunk, the left forearm and upper arm, and the left hand and forearm are denoted θ_1^l, θ_2^l, and θ_3^l, respectively; the right-side angles are θ_1^r, θ_2^r, and θ_3^r. The COM-to-joint ratios for the left upper arm, left forearm, and left hand measured from the shoulder, elbow, and wrist are ρ_1^l, ρ_2^l, and ρ_3^l, respectively; the right-side ratios are ρ_1^r, ρ_2^r, and ρ_3^r. Numerical values are taken from De Leva (De Leva, 1996).

The arm swing is simplified as planar motion in a Cartesian coordinate system, and the Lagrangian for each segment is formulated accordingly, as follows:

$$x_1^l = \rho_1^l l_1^l \cos\theta_1^l. \tag{8}$$

$$y_1^l = -\rho_1^l l_1^l \sin\theta_1^l. \tag{9}$$

$$x_1^r = \rho_1^r l_1^r \cos\theta_1^r. \tag{10}$$

$$y_1^r = -\rho_1^r l_1^r \sin\theta_1^r. \tag{11}$$

$$x_2^l = l_1^l \cos\theta_1^l - \rho_2^l l_2^l \cos(\theta_1^l + \theta_2^l). \tag{12}$$

$$y_2^l = -l_1^l \sin\theta_1^l + \rho_2^l l_2^l \sin(\theta_1^l + \theta_2^l). \tag{13}$$

$$x_2^r = l_1^r \cos\theta_1^r - \rho_2^r l_2^r \cos(\theta_1^r + \theta_2^r). \tag{14}$$

$$y_2^r = -l_1^r \sin\theta_1^r + \rho_2^r l_2^r \sin(\theta_1^r + \theta_2^r). \tag{15}$$

$$x_3^l = l_1^l \cos\theta_1^l - \rho_2^l l_2^l \cos(\theta_1^l + \theta_2^l) + \rho_3^l l_3^l \cos(\theta_1^l + \theta_2^l + \theta_3^l) \tag{16}$$

$$y_3^l = -l_1^l \sin\theta_1^l + \rho_2^l l_2^l \sin(\theta_1^l + \theta_2^l) - \rho_3^l l_3^l \sin(\theta_1^l + \theta_2^l + \theta_3^l) \tag{17}$$

$$x_3^r = l_1^r \cos\theta_1^r - \rho_2^r l_2^r \cos(\theta_1^r + \theta_2^r) + \rho_3^r l_3^r \cos(\theta_1^r + \theta_2^r + \theta_3^r) \tag{18}$$

$$y_3^r = -l_1^r \sin\theta_1^r + \rho_2^r l_2^r \sin(\theta_1^r + \theta_2^r) - \rho_3^r l_3^r \sin(\theta_1^r + \theta_2^r + \theta_3^r). \tag{19}$$

From the above equations, it follows that the Lagrangian expressions for the x- and y-coordinates of the COM of the left and right upper arms, forearms, and hands are separately constructed, thereby facilitating the subsequent computation of the overall Lagrangian for each upper limb.

Based on Lagrangian mechanics(Brizard, 2014), the equations of motion for upper limb movement are given by the Euler-Lagrange equations:

$$\begin{cases} \mathcal{L} = T - V \\ T = T_1^l + T_2^l + T_3^l + T_1^r + T_2^r + T_3^r \\ V = V_1^l + V_2^l + V_3^l + V_1^r + V_2^r + V_3^r \end{cases} . \tag{20}$$

In these expressions, $\mathcal{L}$ denotes the Lagrangian, T the kinetic energy, and V the potential energy. T_1^1, T_2^1, T_3^1 denote the kinetic energies of the left upper arm, left forearm, and left hand, respectively; T_1^r, T_2^r, T_3^rdenote the kinetic energies of the right upper arm, right forearm, and right hand, respectively. V_1^1, V_2^1, V_3^1 denote the potential energies of the left upper arm, left forearm, and left hand, respectively; V_1^r, V_2^r, V_3^r denote the potential energies of the right upper arm, right forearm, and right hand, respectively.

Substituting the per-segment Lagrangians into equations (20) yields:

$$\begin{cases} \mathcal{L}^l = \frac{1}{2}\left(m_1^l {\dot{x}_1^l}^2 + m_1^l {\dot{y}_1^l}^2 + m_2^l {\dot{x}_2^l}^2 + m_2^l {\dot{y}_2^l}^2 + m_3^l {\dot{x}_3^l}^2 + m_3^l {\dot{y}_3^l}^2\right) + \\ \qquad \frac{1}{2}\left(I_1^l {\dot{\theta}_1^l}^2 + I_2^l \left(\dot{\theta}_1^l + \dot{\theta}_2^l\right)^2 + I_3^l \left(\dot{\theta}_1^l + \dot{\theta}_2^l + \dot{\theta}_3^l\right)^2\right) - \\ \qquad g\left(m_1^l y_1^l + m_2^l y_2^l + m_3^l y_3^l\right) \\ \mathcal{L}^r = \frac{1}{2}\left(m_1^r {\dot{x}_1^r}^2 + m_1^r {\dot{y}_1^r}^2 + m_2^r {\dot{x}_2^r}^2 + m_2^r {\dot{y}_2^r}^2 + m_3^r {\dot{x}_3^r}^2 + m_3^r {\dot{y}_3^r}^2\right) + \\ \qquad \frac{1}{2}\left(I_1^r {\dot{\theta}_1^r}^2 + \left(\dot{\theta}_1^r + \dot{\theta}_2^r\right)^2 + I_3^r \left(\dot{\theta}_1^r + \theta_2^r + \dot{\theta}_3^r\right)^2\right) - \\ \qquad g\left(m_1^r y_1^r + m_2^r y_2^r + m_3^r y_3^r\right). \end{cases} \tag{21}$$

In these expressions, $\mathcal{L}^l$ and $\mathcal{L}^r$ denote the Lagrangians of the left and right upper limbs, respectively. m_1^l, m_2^l, m_3^l denote the masses of the left upper arm, left forearm and left hand, respectively; m_1^r, m_2^r, m_3^r denote the masses of the right upper arm, right forearm and right hand, respectively. I_1^l, I_2^l, I_3^l denote the moments of inertia of the left upper arm, left forearm and left hand, respectively; I_1^r, I_2^r, I_3^r denote the moments of inertia of the right upper arm, right forearm and right hand, respectively.

From equation (21) the Lagrangian of the subject's upper limbs during running is obtained, which is then used to perform a quantitative assessment of arm-swing mechanical efficiency.

Over a stride cycle, let index i enumerate the segments (upper arm, forearm and hand). Use the relative angular displaments between adjacent segments, θ_i, as the generalized coordinates for the upper limb Lagrangian, and denote the generalized velocities by $\dot{\theta}_i$. Let t denote time (time is expressed as a percentage, i.e., normalized, so that stance-phase durations are comparable across different gait trials). In combination with lower limb segment quantities that are normalized by body weight, this permits standardized Lagrangian analysis across subjects and gaits. Combining equation (20) with (21) yields the following governing equations:

$$T_1^l = \frac{1}{2} m_1^l \left(\rho_1^l l_1^l \dot{\theta}_1^l\right)^2 + \frac{1}{2} I_1^l \dot{\theta}_1^{l^2}. \tag{22}$$

$$V_1^l = -m_1^l g\left(\rho_1^l l_1^l \sin\theta_1^l\right). \tag{23}$$

$$T_1^r = \frac{1}{2} m_1^r \left(\rho_1^r l_1^r \dot{\theta}_1^r\right)^2 + \frac{1}{2} I_1^r \dot{\theta}_1^{r^2}. \tag{24}$$

$$V_1^r = -m_1^r g\left(\rho_1^r l_1^r \sin\theta_1^r\right). \tag{25}$$

$$T_2^l = \left(m_2^l \left(l_1^l\right)^2 + m_2^l \left(\rho_2^l l_2^l\right)^2 - 2 m_2^l l_1^l \rho_2^l l_2^l \cos\theta_2^l + \frac{1}{2} I_2^l\right) \left(\dot{\theta}_1^{l^2} + \dot{\theta}_2^{l^2} + 2\dot{\theta}_1^l \dot{\theta}_2^l\right). \tag{26}$$

$$V_2^l = -m_2^l g\left(-l_1^l \sin\theta_1^l + \rho_2^l l_2^l \sin\left(\theta_1^l + \theta_2^l\right)\right). \tag{27}$$

$$T_2^r = \left(m_2^r (l_1^r)^2 + m_2^r (\rho_2^r l_2^r)^2 - 2 m_2^r l_1^r \rho_2^r l_2^r \cos\theta_2^r + \frac{1}{2} I_2^r\right) \left(\dot{\theta}_1^{r^2} + \dot{\theta}_2^{r^2} + 2\dot{\theta}_1^r \dot{\theta}_2^r\right). \tag{28}$$

$$V_2^r = -m_2^r g\left(-l_1^r \sin\theta_1^r + \rho_2^r l_2^r \sin(\theta_1^r + \theta_2^r)\right). \tag{29}$$

Compute the distance from the wrist joint to the shoulder; denote the left- and right-side distances by l_w^l and l_w^r, respectively, which are obtained from the following equations:

$$(l_w^l)^2 = l_1^{l^2} + \rho_2^l l_2^{l^2} - 2 l_1^l l_2^l \cos\theta_2^l. \tag{30}$$

$$(l_w^r)^2 = l_1^{r^2} + \rho_2^r l_2^{r^2} - 2 l_1^r l_2^r \cos\theta_2^r. \tag{31}$$

From these distances compute the angle between the forearm and the rotation radius from the wrist to the shoulder. The left- and right-side angles are denoted by θ_w^l and θ_w^r, respectively.

$$\theta_w^l = \arccos\left(\frac{l_1^{l^2} - l_1^l l_2^l \cos\theta_2^l}{l_1^l \left(\sqrt{l_1^{l^2} + \rho_2^l l_2^{l^2} - 2 l_1^l l_2^l \cos\theta_2^l}\right)}\right). \tag{32}$$

$$\theta_w^r = \arccos\left(\frac{{l_1^r}^2 - l_1^r l_2^r \cos\theta_2^r}{l_1^r\left(\sqrt{{l_1^r}^2 + \rho_2^r {l_2^r}^2 - 2 l_1^r l_2^r \cos\theta_2^r}\right)}\right). \tag{33}$$

$$T_3^l = \begin{pmatrix} m_3^l (l_w^l)^2 + m_3^l\left(\rho_3^l l_3^l\right)^2 - \\ 2 m_3^l l_w^l \rho_3^l l_3^l \cos\left(\theta_3^l - \theta_w^l\right) + \frac{1}{2} I_3^l \end{pmatrix}$$
$$\left({\dot\theta_1^l}^2 + {\dot\theta_2^l}^2 + {\dot\theta_2^l}^2 + 2\dot\theta_1^l\dot\theta_2^l + 2\dot\theta_1^l\dot\theta_3^l + 2\dot\theta_2^l\dot\theta_3^l\right). \tag{34}$$

$$V_3^l = -m_3^l g\begin{pmatrix} l_1^l \cos\theta_1^l - \rho_2^l l_2^l \cos\left(\theta_1^l + \theta_2^l\right) + \\ \rho_3^l l_3^l \cos\left(\theta_1^l + \theta_2^l + \theta_3^l\right) \end{pmatrix}. \tag{35}$$

$$T_3^r = \begin{pmatrix} m_3^r (l_w^r)^2 + m_3^r(\rho_3^r l_3^r)^2 - \\ 2 m_3^r l_w^r \rho_3^r l_3^r \cos(\theta_3^r - \theta_w^r) + \frac{1}{2} I_3^r \end{pmatrix}$$
$$\left({\dot\theta_1^r}^2 + {\dot\theta_2^r}^2 + {\dot\theta_3^r}^2 + 2\dot\theta_1^r\dot\theta_2^r + 2\dot\theta_1^r\dot\theta_3^r + 2\dot\theta_2^r\dot\theta_3^r\right). \tag{36}$$

$$V_3^r = -m_3^r g\begin{pmatrix} -l_1^r \sin\theta_1^r + \\ \rho_2^r l_2^r \sin(\theta_1^r + \theta_2^r) - \rho_3^r l_3^r \sin(\theta_1^r + \theta_2^r + \theta_3^r) \end{pmatrix}. \tag{37}$$

Using equations (22) through (26) to compute the kinetic and potential energies of the upper limb, it is found that over a stride cycle $\sum_0^T V(t) = 0$; therefore, only the variations in kinetic energy need to be analyzed.

1.3 Lagrangian Equations of the Lower Limbs

During the flight phase, the form of the lower limb Lagrangian is identical to that used for the upper limbs; one only replaces the masses, COM locations and other segmental parameters with the corresponding lower limb values. Segmental inertial parameters for the lower limb are taken from De Leva (1996). During the stance phase, however, the change in boundary conditions requires re-formulation of the lower limb Lagrangian to account for ground-contact constraints. As the inertial parameters of the left and right lower limbs are identical (De Leva, 1996), a single set of Lagrangian equations suffices to represent either left foot or right foot stance.

Based on the structural characteristics, the Lagrangian equations of the lower limbs are:

$$\begin{cases} \mathcal{L} = T - V \\ T = T_1 + T_2 + T_3 + T_4 + T_5 + T_6 \\ V = V_1 + V_2 + V_3 + V_4 + V_5 + V_6 \end{cases}. \tag{38}$$

In these expressions, $\mathcal{L}$ denotes the Lagrangian, T the kinetic energy, and V the potential energy. T_1, T_2, T_3 denote the kinetic energies of the left foot, left shank and left thigh, respectively; T_4, T_5, T_6 denote the kinetic energies of the right thigh, right shank and right foot, respectively. V_1, V_2, V_3 denote the potential energies of the left foot, left shank and left thigh, respectively; V_4, V_5, V_6 denote the potential energies of the right thigh, right shank and right foot, respectively.

When the subject has reached the target treadmill speed, running becomes a sequence of similar, repeated stride cycles. Hence gait mechanics may be reduced to an analysis over a single stride cycle. Because the net work performed by gravity and the ground reaction force at the beginning and end of a

stride is approximately zero, running can be treated as being governed solely by conservative forces over the cycle. Therefore, the total time derivative of the lower limb Lagrangian can be evaluated.

First, we define the generalized coordinates of the lower limb system. At initial contact the left toe position is (x_0, y_0, z_0); the left ankle (x_1, y_1, z_1); the left knee (x_2, y_2, z_2); the hip positions (x_3, y_3, z_3); the right knee (x_4, y_4, z_4); the right ankle (x_5, y_5, z_5); and the right toe (x_6, y_6, z_6). Segment lengths are denoted as follows: left toe to left ankle l_F^l, left ankle to left knee l_k^l, left knee to left hip l_H^r; the left hip to right hip coordinate is (x_{lr}, y_{lr}, z_{lr}); right hip to right knee l_H^r, right knee to right ankle l_K^r, and right toe to right ankle l_F^r. The distances from joint to segment centre of mass are denoted by d_S^l (left ankle to left shank COM), d_T^l (left knee to left thigh COM), d_T^r (right knee to right thigh COM), and d_S^r (right ankle to right shank COM).

Projecting the lower limb motion onto the sagittal plane and further simplifying the model according to its structural characteristics, the following equalities hold for the joint relative rotation points:

$$(x_0, z_0) = \left(-l_F^l \cos\theta_1, l_F^l \sin\theta_1\right). \tag{39}$$

$$(x_1, z_1) = \left(x_0 + l_K^l \cos(\theta_2 - \theta_1), z_0 + l_K^l \sin(\theta_2 - \theta_1)\right). \tag{40}$$

$$(x_2, z_2) = \begin{pmatrix} x_1 - l_H^l \cos(\theta_3 - \theta_2 + \theta_1), \\ z_1 + l_H^l \sin(\theta_3 - \theta_2 + \theta_1) \end{pmatrix}. \tag{41}$$

$$(x_3, z_3) = (x_2, z_2). \tag{42}$$

$$(x_4, z_4) = \begin{pmatrix} x_3 + l_H^r \cos(\theta_4 + \theta_3 - \theta_2 + \theta_1), \\ z_3 - l_H^r \sin(\theta_4 + \theta_3 - \theta_2 + \theta_1) \end{pmatrix}. \tag{43}$$

$$(x_5, z_5) = \begin{pmatrix} x_4 - l_K^r \cos(\theta_5 - \theta_4 - \theta_3 + \theta_2 - \theta_1), \\ z_4 - l_K^r \sin(\theta_5 - \theta_4 - \theta_3 + \theta_2 - \theta_1) \end{pmatrix}. \tag{44}$$

$$(x_6, z_6) = \begin{pmatrix} x_5 + l_F^r \cos(\theta_6 - \theta_5 + \theta_4 + \theta_3 - \theta_2 + \theta_1), \\ z_5 - l_F^r \sin(\theta_6 - \theta_5 + \theta_4 + \theta_3 - \theta_2 + \theta_1) \end{pmatrix}. \tag{45}$$

For the relative inertial reference frames the following relations hold:

$$(x_0, z_0) = \left(-l_F^l \cos\theta_1, l_F^l \sin\theta_1\right). \tag{46}$$

$$(x_1, z_1) = \begin{pmatrix} -l_F^l \cos\theta_1 + l_K^l \cos(\theta_2 - \theta_1), \\ l_F^l \sin\theta_1 + l_K^l \sin(\theta_2 - \theta_1) \end{pmatrix}. \tag{47}$$

$$(x_2, z_2) = \begin{pmatrix} -l_F^l \cos\theta_1 + l_K^l \cos(\theta_2 - \theta_1) - \\ l_H^l \cos(\theta_3 - \theta_2 + \theta_1), \\ l_F^l \sin\theta_1 + l_K^l \sin(\theta_2 - \theta_1) + \\ l_H^l \sin(\theta_3 - \theta_2 + \theta_1) \end{pmatrix}. \tag{48}$$

$$(x_3, z_3) = (x_2, z_2). \tag{49}$$

$$(x_4, z_4) = \begin{pmatrix} -l_F^l \cos\theta_1 + l_K^l \cos(\theta_2 - \theta_1) - \\ l_H^l \cos(\theta_3 - \theta_2 + \theta_1) + \\ l_H^r \cos(\theta_4 + \theta_3 - \theta_2 + \theta_1), \\ l_F^l \sin\theta_1 + l_K^l \sin(\theta_2 - \theta_1) + \\ l_H^l \sin(\theta_3 - \theta_2 + \theta_1) - \\ l_H^r \sin(\theta_4 + \theta_3 - \theta_2 + \theta_1) \end{pmatrix}. \tag{50}$$

$$(x_5, z_5) = \begin{pmatrix} -l_F^l \cos\theta_1 + l_K^l \cos(\theta_2 - \theta_1) - \\ l_H^l \cos(\theta_3 - \theta_2 + \theta_1) + \\ l_H^r \cos(\theta_4 + \theta_3 - \theta_2 + \theta_1) - \\ l_K^r \cos(\theta_5 - \theta_4 - \theta_3 + \theta_2 - \theta_1), \\ l_F^l \sin\theta_1 + l_K^l \sin(\theta_2 - \theta_1) + \\ l_H^l \sin(\theta_3 - \theta_2 + \theta_1) - \\ l_H^r \sin(\theta_4 + \theta_3 - \theta_2 + \theta_1) - \\ l_K^r \sin(\theta_5 - \theta_4 - \theta_3 + \theta_2 - \theta_1) \end{pmatrix}. \tag{51}$$

$$(x_6, z_6) = \begin{pmatrix} -l_F^l \cos\theta_1 + l_K^l \cos(\theta_2 - \theta_1) - \\ l_H^l \cos(\theta_3 - \theta_2 + \theta_1) + \\ l_H^r \cos(\theta_4 + \theta_3 - \theta_2 + \theta_1) - \\ l_K^r \cos(\theta_5 - \theta_4 - \theta_3 + \theta_2 - \theta_1) + \\ l_F^r \cos(\theta_6 - \theta_5 + \theta_4 + \theta_3 - \theta_2 + \theta_1), \\ l_F^l \sin\theta_1 + l_K^l \sin(\theta_2 - \theta_1) + \\ l_H^l \sin(\theta_3 - \theta_2 + \theta_1) - \\ l_H^r \sin(\theta_4 + \theta_3 - \theta_2 + \theta_1) - \\ l_K^r \sin(\theta_5 - \theta_4 - \theta_3 + \theta_2 - \theta_1) - \\ l_F^r \sin(\theta_6 - \theta_5 + \theta_4 + \theta_3 - \theta_2 + \theta_1) \end{pmatrix}. \tag{52}$$

When a segmental COM is expressed with respect to the relative inertial reference frame, the following relations hold:

$$(x_F^l, z_F^l) = (-d_K^l \cos\theta_1, d_K^l \sin\theta_1). \tag{53}$$

$$(x_S^l, z_S^l) = \begin{pmatrix} -l_F^l \cos\theta_1 + d_H^l \cos(\theta_2 - \theta_1), \\ l_F^l \sin\theta_1 + d_H^l \sin(\theta_2 - \theta_1) \end{pmatrix}. \tag{54}$$

$$(x_T^l, z_T^l) = \begin{pmatrix} -l_F^l \cos\theta_1 + l_K^l \cos(\theta_2 - \theta_1) - \\ d_H^l \cos(\theta_3 - \theta_2 + \theta_1), \\ l_F^l \sin\theta_1 + l_K^l \sin(\theta_2 - \theta_1) + \\ d_H^l \sin(\theta_3 - \theta_2 + \theta_1) \end{pmatrix}. \tag{55}$$

$$(x_T^r, z_T^r) = \begin{pmatrix} -l_F^l \cos\theta_1 + l_K^l \cos(\theta_2 - \theta_1) - \\ l_H^l \cos(\theta_3 - \theta_2 + \theta_1) + \\ (l_H^r - d_H^r) \cos(\theta_4 + \theta_3 - \theta_2 + \theta_1), \\ l_F^l \sin\theta_1 + l_K^l \sin(\theta_2 - \theta_1) + \\ l_H^l \sin(\theta_3 - \theta_2 + \theta_1) - \\ (l_H^r - d_H^r) \sin(\theta_4 + \theta_3 - \theta_2 + \theta_1) \end{pmatrix}. \tag{56}$$

$$(x_S^r, z_S^r) = \begin{pmatrix} -l_F^l\cos\theta_1 + l_K^l\cos(\theta_2 - \theta_1) - \\ l_H^l\cos\,(\theta_3 - \theta_2 + \theta_1) + \\ l_H^r\cos(\theta_4 + \theta_3 - \theta_2 + \theta_1) - \\ (l_K^r - d_K^r)\cos(\theta_5 - \theta_4 - \theta_3 + \theta_2 - \theta_1), \\ l_F^l\sin\theta_1 + l_K^l\sin(\theta_2 - \theta_1) + \\ l_H^l\sin\,(\theta_3 - \theta_2 + \theta_1) - \\ l_H^r\sin(\theta_4 + \theta_3 - \theta_2 + \theta_1) - \\ (l_K^r - d_K^r)\sin(\theta_5 - \theta_4 - \theta_3 + \theta_2 - \theta_1) \end{pmatrix}. \tag{57}$$

$$(x_F^r, z_F^r) = \begin{pmatrix} -l_F^l\cos\theta_1 + l_K^l\cos(\theta_2 - \theta_1) - \\ l_H^l\cos\,(\theta_3 - \theta_2 + \theta_1) + \\ l_H^r\cos(\theta_4 + \theta_3 - \theta_2 + \theta_1) - \\ l_K^r\cos(\theta_5 - \theta_4 - \theta_3 + \theta_2 - \theta_1) + \\ (l_F^r - d_F^r)\cos(\theta_6 - \theta_5 + \theta_4 + \theta_3 - \theta_2 + \theta_1), \\ l_F^l\sin\theta_1 + l_K^l\sin(\theta_2 - \theta_1) + \\ l_H^l\sin\,(\theta_3 - \theta_2 + \theta_1) - \\ l_H^r\sin(\theta_4 + \theta_3 - \theta_2 + \theta_1) - \\ l_K^r\sin(\theta_5 - \theta_4 - \theta_3 + \theta_2 - \theta_1) - \\ (l_F^r - d_F^r)\sin(\theta_6 - \theta_5 + \theta_4 + \theta_3 - \theta_2 + \theta_1) \end{pmatrix}. \tag{58}$$

During the stance phase, take the intersegmental angular displacements θ_i between adjacent limb segments as the generalized coordinates of the lower limb Lagrangian, and denote the generalized velocities by $\dot{\theta}_i$. Time t is expressed as a percentage of the stance period (i.e., time normalised) to permit comparability of stance durations across different gaits. Combined with body weight normalization of both upper and lower limb segmental quantities, taking the total time derivative of the lower limb Lagrangian yields:

$$\frac{d}{dt}\left(\frac{\vartheta\mathcal{L}}{\vartheta\dot{\theta}_i}\right) - \left(\frac{\vartheta\mathcal{L}}{\vartheta\theta_i}\right) = 0. \tag{59}$$

Using the coordinates of the segmental relative inertial reference frames and setting $(x_0, z_0) = (0, 0)$, and by substituting the relations between the joint-relative inertial frames and the segment-COM relative inertial frames, the total-time-derivative form of the lower limb Lagrangian produces explicit expressions for the segmental kinetic and potential energies:

$$T_1 = \frac{1}{2}\left(m_1\left(\left(-d_K^l\cos\theta_1\right)^2 + \left(d_K^l\sin\theta_1\right)^2\right) + I_1\right)\dot{\theta}_1^2. \tag{60}$$

$$V_1 = m_1 g\left(d_K^l\sin\theta_1\right). \tag{61}$$

$$T_2 = \frac{1}{2}\left(m_2\begin{pmatrix}\left(-l_F^l\cos\theta_1 + d_H^l\cos(\theta_2 - \theta_1)\right)^2 + \\ \left(l_F^l\sin\theta_1 + d_H^l\sin(\theta_2 - \theta_1)\right)^2\end{pmatrix} + I_2\right). \left(\dot{\theta}_2^2 + \dot{\theta}_1^2 + 2\dot{\theta}_2\dot{\theta}_1\right) \tag{62}$$

$$V_2 = m_2 g\left(l_F^l\sin\theta_1 + d_H^l\sin(\theta_2 - \theta_1)\right). \tag{63}$$

$$T_3 = \frac{1}{2}\left(m_3 \left(\begin{pmatrix} -l_F^l \cos\theta_1 + l_K^l \cos(\theta_2 - \theta_1) - \\ d_H^l \cos(\theta_3 - \theta_2 + \theta_1) \end{pmatrix}^2 + \begin{pmatrix} l_F^l \sin\theta_1 + l_K^l \sin(\theta_2 - \theta_1) + \\ d_H^l \sin(\theta_3 - \theta_2 + \theta_1) \end{pmatrix}^2 \right) + I_3 \right)$$

$$\left(\dot{\theta}_3^2 + \dot{\theta}_2^2 + \dot{\theta}_1^2 + 2\dot{\theta}_3\dot{\theta}_2 + 2\dot{\theta}_3\dot{\theta}_1 + 2\dot{\theta}_2\dot{\theta}_1\right). \tag{64}$$

$$V_3 = m_3 g \begin{pmatrix} l_F^l \sin\theta_1 + l_K^l \sin(\theta_2 - \theta_1) + \\ d_H^l \sin(\theta_3 - \theta_2 + \theta_1) \end{pmatrix}. \tag{65}$$

$$T_4 = \frac{1}{2}\left(m_4 \left(\begin{pmatrix} -l_F^l \cos\theta_1 + l_K^l \cos(\theta_2 - \theta_1) - \\ l_H^l \cos(\theta_3 - \theta_2 + \theta_1) + \\ (l_H^r - d_H^r)\cos(\theta_4 + \theta_3 - \theta_2 + \theta_1) \end{pmatrix}^2 + \begin{pmatrix} l_F^l \sin\theta_1 + l_K^l \sin(\theta_2 - \theta_1) + \\ l_H^l \sin(\theta_3 - \theta_2 + \theta_1) - \\ (l_H^r - d_H^r)\sin(\theta_4 + \theta_3 - \theta_2 + \theta_1) \end{pmatrix}^2 \right) + I_4 \right)$$

$$\begin{pmatrix} \dot{\theta}_4^2 + \dot{\theta}_3^2 + \dot{\theta}_2^2 + \dot{\theta}_1^2 + 2\dot{\theta}_4\dot{\theta}_3 + 2\dot{\theta}_4\dot{\theta}_2 + \\ 2\dot{\theta}_4\dot{\theta}_1 + 2\dot{\theta}_3\dot{\theta}_2 + 2\dot{\theta}_3\dot{\theta}_1 + 2\dot{\theta}_2\dot{\theta}_1 \end{pmatrix}. \tag{66}$$

$$V_4 = m_4 g \begin{pmatrix} l_F^l \sin\theta_1 + l_K^l \sin(\theta_2 - \theta_1) + \\ l_H^l \sin(\theta_3 - \theta_2 + \theta_1) - \\ (l_H^r - d_H^r)\sin(\theta_4 + \theta_3 - \theta_2 + \theta_1) \end{pmatrix}. \tag{67}$$

$$T_5 = \frac{1}{2}\left(m_5 \left(\begin{pmatrix} -l_F^l \cos\theta_1 + l_K^l \cos(\theta_2 - \theta_1) - l_H^l \cos(\theta_3 - \theta_2 + \theta_1) + \\ l_H^r \cos(\theta_4 + \theta_3 - \theta_2 + \theta_1) - (l_K^r - d_K^r)\cos(\theta_5 - \theta_4 - \theta_3 + \theta_2 - \theta_1) \end{pmatrix}^2 + \begin{pmatrix} l_F^l \sin\theta_1 + l_K^l \sin(\theta_2 - \theta_1) + l_H^l \sin(\theta_3 - \theta_2 + \theta_1) - \\ l_H^r \sin(\theta_4 + \theta_3 - \theta_2 + \theta_1) - (l_K^r - d_K^r)\sin(\theta_5 - \theta_4 - \theta_3 + \theta_2 - \theta_1) \end{pmatrix}^2 \right) + I_5 \right)$$

$$\begin{pmatrix} \dot{\theta}_5^2 + \dot{\theta}_4^2 + \dot{\theta}_3^2 + \dot{\theta}_2^2 + \dot{\theta}_1^2 + 2\dot{\theta}_5\dot{\theta}_4 + 2\dot{\theta}_5\dot{\theta}_3 + 2\dot{\theta}_5\dot{\theta}_2 + 2\dot{\theta}_5\dot{\theta}_1 + \\ 2\dot{\theta}_4\dot{\theta}_3 + 2\dot{\theta}_4\dot{\theta}_2 + 2\dot{\theta}_4\dot{\theta}_1 + 2\dot{\theta}_3\dot{\theta}_2 + 2\dot{\theta}_3\dot{\theta}_1 + 2\dot{\theta}_2\dot{\theta}_1 \end{pmatrix}. \tag{68}$$

$$V_5 = m_5 g \begin{pmatrix} l_F^l \sin\theta_1 + l_K^l \sin(\theta_2 - \theta_1) + l_H^l \sin(\theta_3 - \theta_2 + \theta_1) - \\ l_H^r \sin(\theta_4 + \theta_3 - \theta_2 + \theta_1) - (l_K^r - d_K^r)\sin(\theta_5 - \theta_4 - \theta_3 + \theta_2 - \theta_1) \end{pmatrix}. \tag{69}$$

$$T_6 = \frac{1}{2}\left(m_6 \left(\begin{pmatrix} -l_F^l \cos\theta_1 + l_K^l \cos(\theta_2 - \theta_1) - l_H^l \cos(\theta_3 - \theta_2 + \theta_1) + \\ l_H^r \cos(\theta_4 + \theta_3 - \theta_2 + \theta_1) - l_K^r \cos(\theta_5 - \theta_4 - \theta_3 + \theta_2 - \theta_1) + \\ (l_F^r - d_F^r)\cos(\theta_6 - \theta_5 + \theta_4 + \theta_3 - \theta_2 + \theta_1) \end{pmatrix}^2 + \begin{pmatrix} l_F^l \sin\theta_1 + l_K^l \sin(\theta_2 - \theta_1) + l_H^l \sin(\theta_3 - \theta_2 + \theta_1) - \\ l_H^r \sin(\theta_4 + \theta_3 - \theta_2 + \theta_1) - l_K^r \sin(\theta_5 - \theta_4 - \theta_3 + \theta_2 - \theta_1) - \\ (l_F^r - d_F^r)\sin(\theta_6 - \theta_5 + \theta_4 + \theta_3 - \theta_2 + \theta_1) \end{pmatrix}^2 \right) + I_6 \right)$$

$$\begin{pmatrix} \dot{\theta}_6^2 + \dot{\theta}_5^2 + \dot{\theta}_4^2 + \dot{\theta}_3^2 + \dot{\theta}_2^2 + \dot{\theta}_1^2 + 2\dot{\theta}_6\dot{\theta}_5 + 2\dot{\theta}_6\dot{\theta}_4 + 2\dot{\theta}_6\dot{\theta}_3 + 2\dot{\theta}_6\dot{\theta}_2 + 2\dot{\theta}_6\dot{\theta}_1 + \\ 2\dot{\theta}_5\dot{\theta}_4 + 2\dot{\theta}_5\dot{\theta}_3 + 2\dot{\theta}_5\dot{\theta}_2 + 2\dot{\theta}_5\dot{\theta}_1 + 2\dot{\theta}_4\dot{\theta}_3 + 2\dot{\theta}_4\dot{\theta}_2 + 2\dot{\theta}_4\dot{\theta}_1 + 2\dot{\theta}_3\dot{\theta}_2 + 2\dot{\theta}_3\dot{\theta}_1 + 2\dot{\theta}_2\dot{\theta}_1 \end{pmatrix}. \tag{70}$$

$$V_6 = m_6 g \begin{pmatrix} l_F^l \sin\theta_1 + l_K^l \sin(\theta_2 - \theta_1) + l_H^l \sin(\theta_3 - \theta_2 + \theta_1) - l_H^r \sin(\theta_4 + \theta_3 - \theta_2 + \theta_1) - \\ l_K^r \sin(\theta_5 - \theta_4 - \theta_3 + \theta_2 - \theta_1) - (l_F^r - d_F^r)\sin(\theta_6 - \theta_5 + \theta_4 + \theta_3 - \theta_2 + \theta_1) \end{pmatrix}. \quad (71)$$

From equations (60) through (71) the Lagrangian of the subject's lower limb during the stance phase is obtained. During the flight phase the lower-limb Lagrangian is computed from equation (38). Together these expressions permit a quantitative evaluation of lower-limb mechanical energy expenditure over the running stride cycle using the Lagrangian formalism.

2 APPENDIX B

The following describes the MATLAB code used for data processing and analysis. The code reads time-series data from an Excel file, performs a Fourier-series fit on each column of the data, and automatically selects the optimal series order for efficient smoothing. The code was developed in MATLAB (R2021b) and relies on built-in functions; no external packages are required, the main script can be run in MATLAB, specifying the input file if necessary.

Algorithm 1 Data Preprocessing Module

```
[~, ~, raw] = xlsread('data_100.xlsx');

headers = raw(1:2, :);

data = cell2mat(raw(3:end, :));

[n_samples, n_cols] = size(data);

t = (0:n_samples-1)'/(n_samples-1)*2*pi;
```

Explanation:

Read all contents of the Excel file into raw.

headers stores the first two rows of text (e.g., the column headers).

From the third row onward, the data are numeric; use cell2mat to convert them into a pure numeric matrix called data.

n_samples is the number of samples (rows), and n_cols is the number of data columns.

Create a time vector t that ranges from 0 to 2π with n_samples uniformly distributed points (corresponding to (72)).

$$t_i = \frac{i}{n-1} \cdot 2\pi, i = 0,1,\dots,n-1. \tag{72}$$

Purpose: Let t be the standardized time column vector of length nnn, and let i denote the sample index. Each sample is uniformly mapped onto the interval $[0,2\pi]$, so that the data lie on a periodic domain suitable for Fourier analysis.

Algorithm 2 Global Parameter Settings

```
max_k = min(20, floor(n_samples/2));

min_k = 10;

smooth_window = 2;
```

```
thresh_factor = 1.5;
```

max_k: The maximum number of Fourier terms, not exceeding 20 or half the number of data points.

min_k: The minimum number of Fourier terms, at least 10.

smooth_window: The size of the moving window used for smoothing to prevent curve jitter.

thresh_factor: The curvature amplification factor, used to detect inflection points (controls detection sensitivity).

Algorithm 3 Precompute Trigonometric Function Matrix

```
sin_matrix = sin((1:max_k)' * t');

cos_matrix = cos((1:max_k)' * t');
```

Explanation:

Precompute all sine and cosine terms required for an order-k Fourier expansion(corresponding to (73)).

During fitting, use these precomputed values directly to accelerate computation.

$$s(x) \sim A_0 + \sum_{n=1}^{\infty} \left(A_n \cos(\frac{2\pi n x}{p}) + B_n \sin(\frac{2\pi n x}{p}) \right). \tag{73}$$

Purpose:This is the standard form of the Fourier series, which states that any periodic function can be represented as a weighted sum of sine and cosine terms. It is used here for fitting and reconstructing the data.

Algorithm 4 Core Processing Workflow

```
residual_matrix = zeros(max_k, n_cols);

optimal_orders = zeros(n_cols, 1);

color_map = hsv(n_cols);



for col = 1:n_cols

    signal = data(:, col) - mean(data(:, col));    % remove DC component
```

```
    for k = 1:max_k

        X = [ones(n_samples,1), sin_matrix(1:k,:)', cos_matrix(1:k,:)'];

        residual_matrix(k, col) = norm(signal - X * (X\signal)) / sqrt(n_samples);

        residual_matrix(k, col) = norm(signal - X * (X\signal)) / sqrt(n_samples);

    end

  end
```

Explanation:

residual_matrix stores, for each data column and each Fourier order, the fitting residuals.

optimal_orders saves the optimal order for each data column.

color_map provides a distinct color for each data column when plotting.

For each data column: subtract the mean (removing the DC component) to retain only the fluctuating part.

Construct the design matrix X, including a constant term, k sine terms, and k cosine terms, for a total of 2k + 1 columns (corresponding to (74)).

Perform a least-squares fit Xβ ≈ signal to obtain the optimal parameters (corresponding to (75)).

After fitting, compute the residuals RMSE (Root Mean Square Error) (corresponding to (76)).

$$X = \begin{bmatrix} 1 & \sin(t_0) & \dots & \sin(Kt_0) & \cos(t_0) & \dots & \cos(Kt_0) \\ 1 & \sin(t_1) & \dots & \sin(Kt_1) & \cos(t_1) & \dots & \cos(Kt_1) \\ \vdots & \vdots & \ddots & \vdots & \vdots & \ddots & \vdots \\ 1 & \sin(t_{n-1}) & \dots & \sin(Kt_{n-1}) & \cos(t_{n-1}) & \dots & \cos(Kt_{n-1}) \end{bmatrix}. \quad (74)$$

Purpose: Construct the design (regression) matrix used for least-squares fitting, representing the constant term together with the sine and cosine basis functions in matrix form to facilitate solution by linear regression.

$$\hat{\beta} = (X^T X)^{-1} X^T y. \quad (75)$$

Purpose: Apply the least-squares formula to obtain the Fourier-series coefficients A_0, A_n, B_n, thereby fitting the data.

$$\text{RMSD} = \sqrt{\frac{\sum_{t=1}^{T}(\hat{y}_t - y_t)^2}{T}}. \quad (76)$$

Purpose:Measure the fitting error of the model to the original signal under different Fourier orders (i.e., different model complexities). The RMSD values are used to select the optimal order k.

Algorithm 5 Curvature Detection and Automatic Selection of Optimal k

```
log_res   = log(residual_matrix(:, col) * sqrt(n_samples));
smoothed  = movmean(log_res, smooth_window);
delta     = diff(smoothed);
curvature = abs(diff(delta)) ./ (abs(delta(1:end-1)) + 1e-6);
base_level   = median(curvature(1:5));
valid_region = find(curvature > thresh_factor * base_level, 1, 'first');

if ~isempty(valid_region)
    [~, idx] = max(curvature(valid_region:end));
    k_opt = valid_region + idx - 1;
    if (residual_matrix(k_opt, col) / residual_matrix(1, col)) > 0.85
        k_opt = find(residual_matrix(:, col) < 0.7 * residual_matrix(1, col), 1);
    end
else
    k_opt = find(residual_matrix(:, col) < 0.5 * residual_matrix(1, col), 1);
end
k_opt = clamp(k_opt, min_k, max_k);
optimal_orders(col) = k_opt;
```

Explanation:

Take the logarithm of the residual norm and apply smoothing.

Compute the first derivative (delta), then compute the curvature (i.e., the rate of change of the rate of change).

Positions with high curvature correspond to inflection points (where increasing k yields the most significant improvement).

Baseline curvature is defined as the median of the first five points.

Find the first point where the curvature exceeds thresh_factor times the baseline.

If there is a clear inflection point, select the position with the maximum curvature after the inflection (corresponding to (77) and (78)).

If not, use a simple residual drop ratio to determine the optimal order.

Constrain k within the range [min_k, max_k] to prevent it from being too large or too small.

First difference:

$$\varDelta_1 k = \mathrm{RMSD}_{k+1} - \mathrm{RMSD}_k. \tag{77}$$

Second difference:

$$\varDelta_2 k = \varDelta_1(k+1) - \varDelta_1(k). \tag{78}$$

Purpose:Perform first- and second-difference analysis on the residuals to compute the "rate of change of the rate of change" of the fitting error (i.e., the acceleration of fit improvement). This helps identify the inflection point—the optimal choice of Fourier order.

Algorithm 6 Results Visualization

```
subplot(3,1,1)

hold on

for col = 1:n_cols

    plot(1:max_k, residual_matrix(:, col), ...);

    plot(optimal_orders(col), residual_matrix(optimal_orders(col), col), '^', ...)

    text(...)

end


subplot(3,1,2)

stem(optimal_orders, 'filled', 'MarkerSize', 8)


subplot(3,1,3)

hold on
```

```
for col = 1:n_cols
    semilogy(1:max_k, residual_matrix(:, col) * sqrt(n_samples), ...)
end
```

Explanation:

Subplot 1: Plot the residual curves versus Fourier order for each data column. Mark the optimal order with a triangle symbol and annotate it with text. Optionally, add a secondary x-axis at the top to convert the order index into frequency units for easier physical interpretation.

Subplot 2: Show the distribution of optimal orders across data columns using a stem plot, which makes it easy to compare the complexity required for different signals.

Subplot 3: Plot the residual norms. Using a logarithmic scale emphasizes the overall decreasing trend of residuals with increasing order and enhances the visibility of differences across data columns.

3 APPENDIX C

Estimation of Physiological Common Equations and their Calculation Results (Table S1)

ACSM (American College of Sports Medicine, 2013):

$$\dot{V}O_2=3.5+0.2\times speed+0.9+0.9\times speed\times grade. \tag{1}$$

$speed$ in m/min,$grade$ is percent grade expressed in decimal format (e.g., 10% = 0.10)

Weyand (Weyand et al., 2021):

$$\dot{V}O_2=3.05+\frac{(w+l)}{w}t(0.32g+3.28+(1+0.19g)2.66s^2). \tag{2}$$

w = body weight (kg), l = load (kg), t = terrain factor, g = grade (%)—(0 to 100), s = speed (m/s).

Pandolf (Pandolf et al., 1977):

$$\dot{V}O_2=\frac{2.985}{w}\left(1.5w+2(w+l)\left(\frac{l}{w}\right)^2+t(w+l)(1.5s^2+0.35\times s\times g)\right) .\tag{3}$$

w = body weight (kg), l = load (kg), t = terrain factor, s = speed (m/s), g = grade (%)—(0 to 100).

Van der Wal (van der Walt and Wyndham, 1973):

$$\dot{V}O_2\ (L\cdot min^{-1})=-0.419+0.03257M+0.000117MV^2. \tag{4}$$

M = body weight (kg), V = speed (m/s).

Lejeune (Lejeune et al., 1998):

$$\dot{V}O_2=2.209+3.1633v. \tag{5}$$

v = speed (km/h)

Conversions from energy to O_2 units were implemented for the Pandolf and Looney equations using a conversion factor of 20.1 mL of O_2 per Joule per Blaxter (Blaxter, 1989):

$$E\ (kJ)=\dot{V}O_2\ (L\cdot kg^{-1}\cdot min^{-1})\times w\times t\times 20.1. \tag{6}$$

w = body weight (kg), t = duration (min).

$$\text{Energy (kcal)} = \frac{\text{E (KJ)}}{4.184}. \tag{7}$$

Other Supplementary Materials for this manuscript include the following:

Test Report S1 Natural Arm Swing.

Includes 3D pressure plots, gait parameters (step length, stride and step time, stride length, step width, cadence, velocity, and phase percentages), COM (butterfly) analysis, force and pressure curves, force parameters (maximum force left/right and timing), and forefoot–midfoot–heel analyses. This report was obtained using a pressure treadmill (FDM-THQ-M-3i, zebris Medical GmbH, Germany) with a sampling frequency of 300 Hz. Numerical results and figures are provided in the file.

Test Report S2 Modified Arm Swing.

Contains the same types of data, including 3D pressure plots, forefoot/backfoot force distribution, pressure plots, and related gait and force parameters. This report was also obtained using a pressure treadmill (FDM-THQ-M-3i, zebris Medical GmbH, Germany) with a sampling frequency of 300 Hz. Numerical results and figures are provided in the file.

4 References

Agarana, M. C., and Akinlabi, E. T. (2018). Mathematical modelling and analysis of human arm as a triple pendulum system using euler–lagragian model., in *IOP Conference Series: Materials Science and Engineering*, (IOP Publishing)413, 012010. Available at: https://iopscience.iop.org/article/10.1088/1757-899X/413/1/012010/meta (Accessed August 23, 2025).

American College of Sports Medicine (2013). *ACSM's health-related physical fitness assessment manual*. Philadelphia: Lippincott Williams & Wilkins.

Aristidou, A., Cohen-Or, D., Hodgins, J. K., and Shamir, A. (2018). Self-similarity analysis for motion capture cleaning. *Comput. Graph. Forum* 37, 297–309. doi: 10.1111/cgf.13362

Blaxter, K. L. (1989). *Energy metabolism in animals and man*. Cambridge: Cambridge University Press.

Brizard, A. J. (2014). *Introduction to lagrangian mechanics, 2nd Edn*. Singapore: World Scientific Publishing Company.

Camazine, S., Deneubourg, J. L., Theraulaz, G., Sneyd, J., and Franks, N. R. (2020). *Self-organization in biological systems*. Princeton: Princeton University Press.

De Leva, P. (1996). Adjustments to Zatsiorsky-Seluyanov's segment inertia parameters. *J. Biomech.* 29, 1223–1230. doi: 10.1016/0021-9290(95)00178-6

Juarrero, A. (2000). Dynamics in action: Intentional behavior as a complex system. *Emergence* 2, 24–57. doi: 10.1207/S15327000EM0202_03

Lejeune, T. M., Willems, P. A., and Heglund, N. C. (1998). Mechanics and energetics of human locomotion on sand. *J. Exp. Biol.* 201, 2071–2080. doi: 10.1242/jeb.201.13.2071

Li, T., and Yorke, J. A. (1975). Period three implies chaos. *Am. Math. Mon.* 82, 985–992. doi: 10.2307/2318254

Pandolf, K. B., Givoni, B., and Goldman, R. F. (1977). Predicting energy expenditure with loads while standing or walking very slowly. *J. Appl. Physiol.: Respir. Environ. Exerc. Physiol.* 43, 577–581. doi: 10.1152/jappl.1977.43.4.577

Prigogine, I., and Stengers, I. (2018). *Order out of chaos: Man's new dialogue with nature*. London: Verso Books.

van der Walt, W. H., and Wyndham, C. H. (1973). An equation for prediction of energy expenditure of walking and running. *J. Appl. Physiol.* 34, 559–563. doi: 10.1152/jappl.1973.34.5.559

Weyand, P. G., Ludlow, L. W., Nollkamper, J. J., and Buller, M. J. (2021). Real-world walking economy: Can laboratory equations predict field energy expenditure? *J. Appl. Physiol.* 131, 1272–1285. doi: 10.1152/japplphysiol.00121.2021

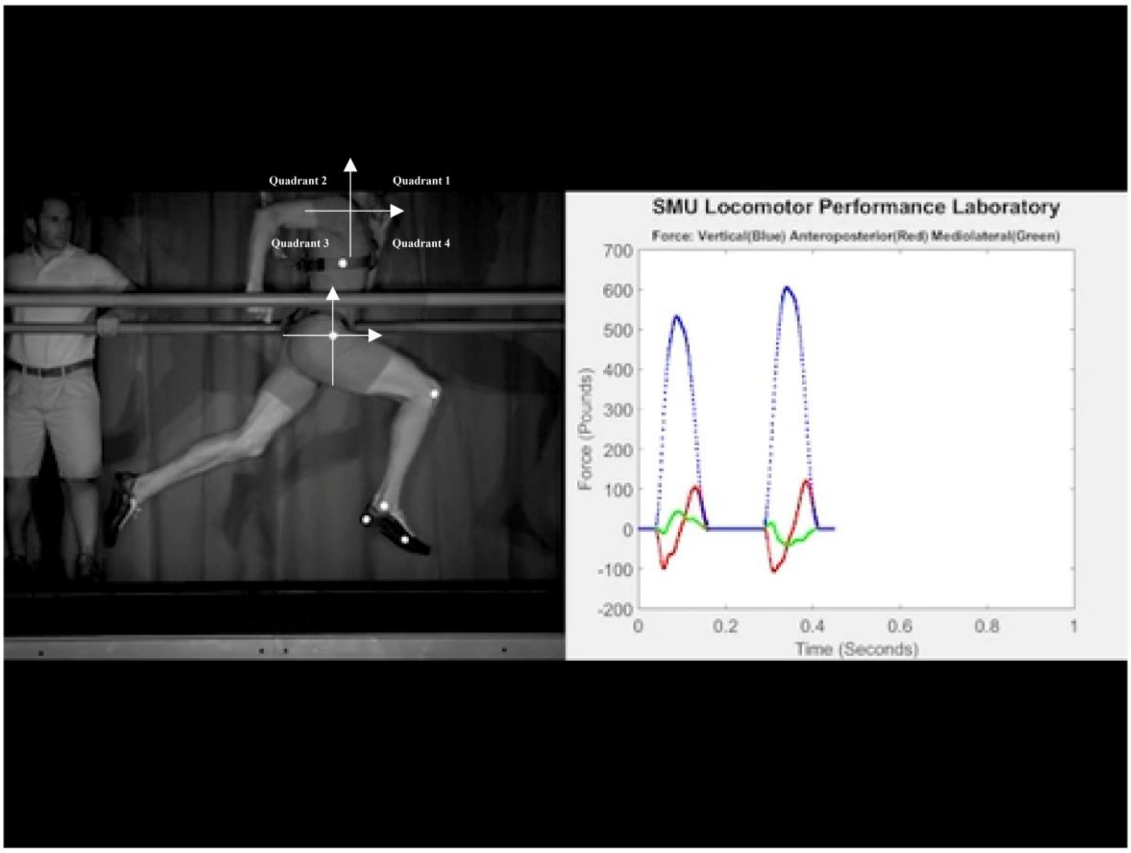

Figure S1. Key video frame used for Wariner-based technical learning and arm–trunk coordination analysis. The shoulder-centered sagittal-plane Cartesian coordinate system is shown, with the shoulder joint defined as the pivot point.

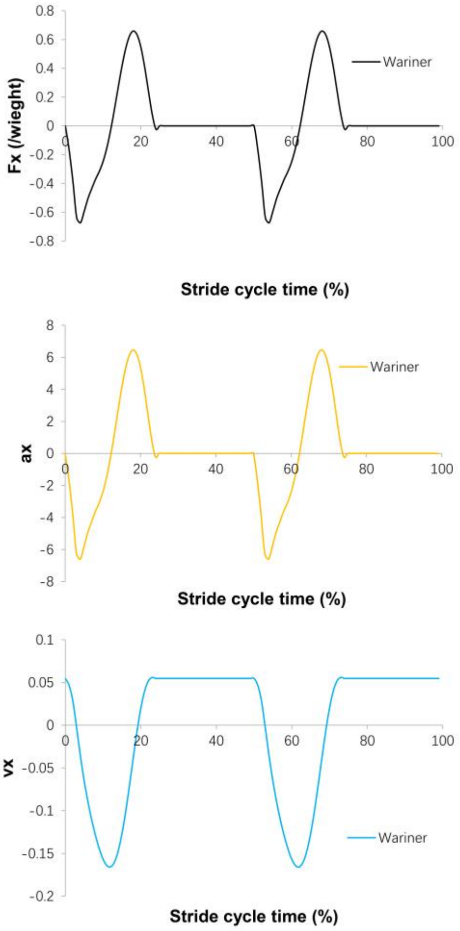

Figure S2. Anterior–posterior GRF, COM acceleration, and COM velocity derived from Wariner's biomechanical data. The GRF was normalized to body weight.

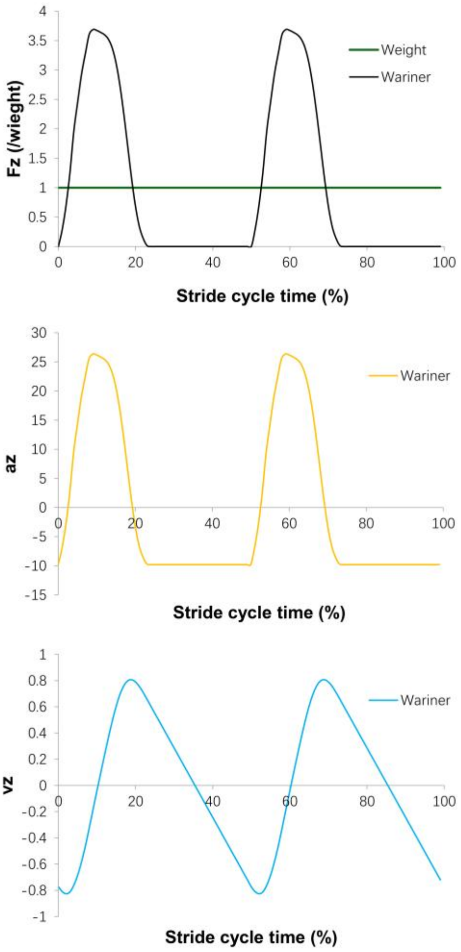

Figure S3. Vertical GRF, COM acceleration, and COM velocity derived from Wariner's biomechanical data. The vertical GRF was normalized to body weight.

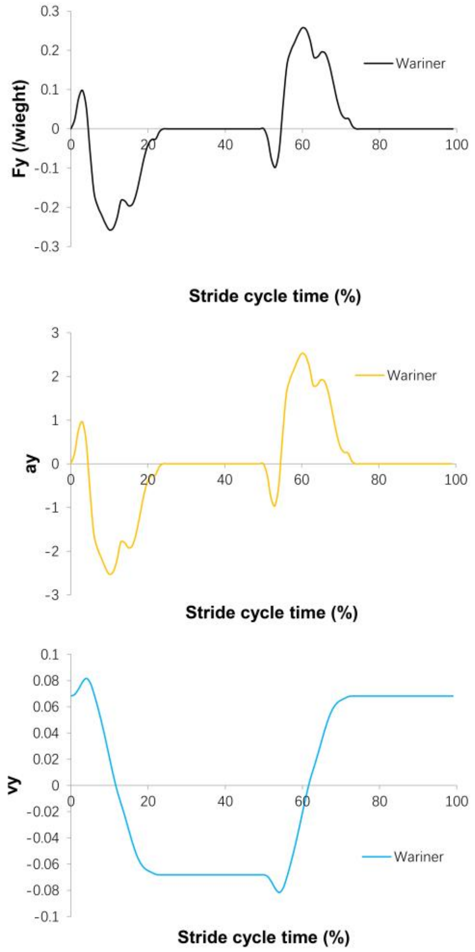

Figure S4. Mediolateral GRF, COM acceleration, and COM velocity derived from Wariner's biomechanical data. The GRF was normalized to body weight.

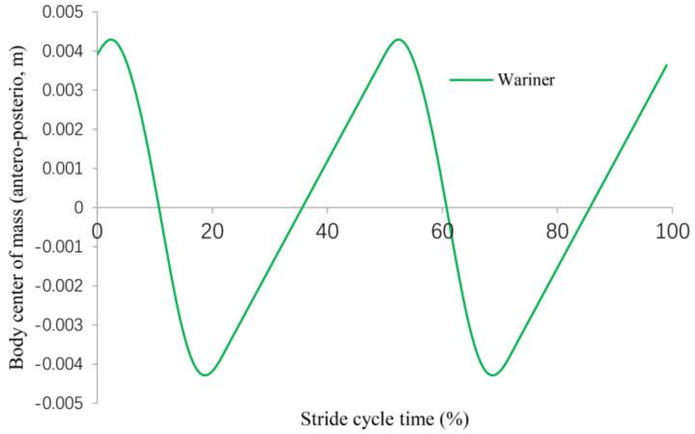

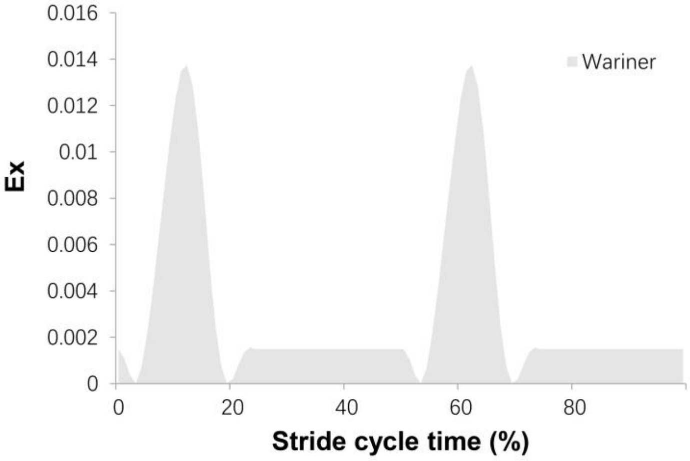

Figure S5. Anterior–posterior COM displacement and mechanical energy-related variable derived from Wariner's biomechanical data.

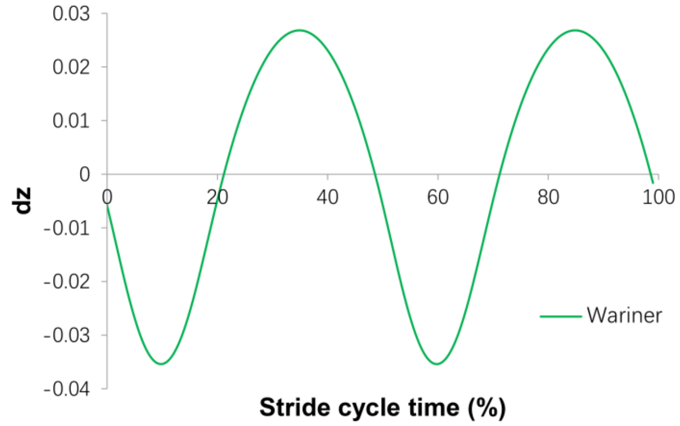

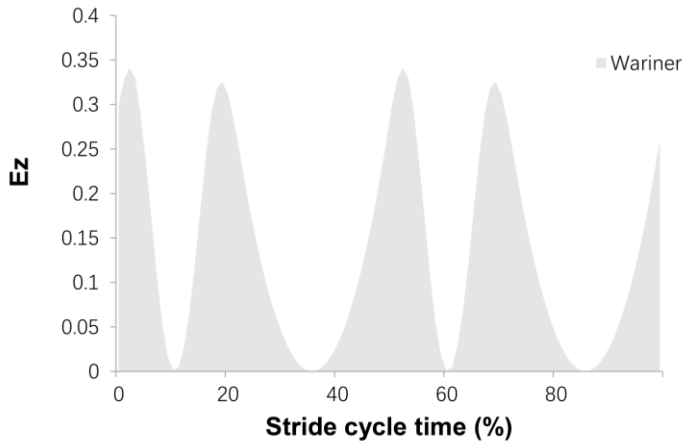

Figure S6. Vertical COM displacement and mechanical energy-related variable derived from Wariner's biomechanical data.

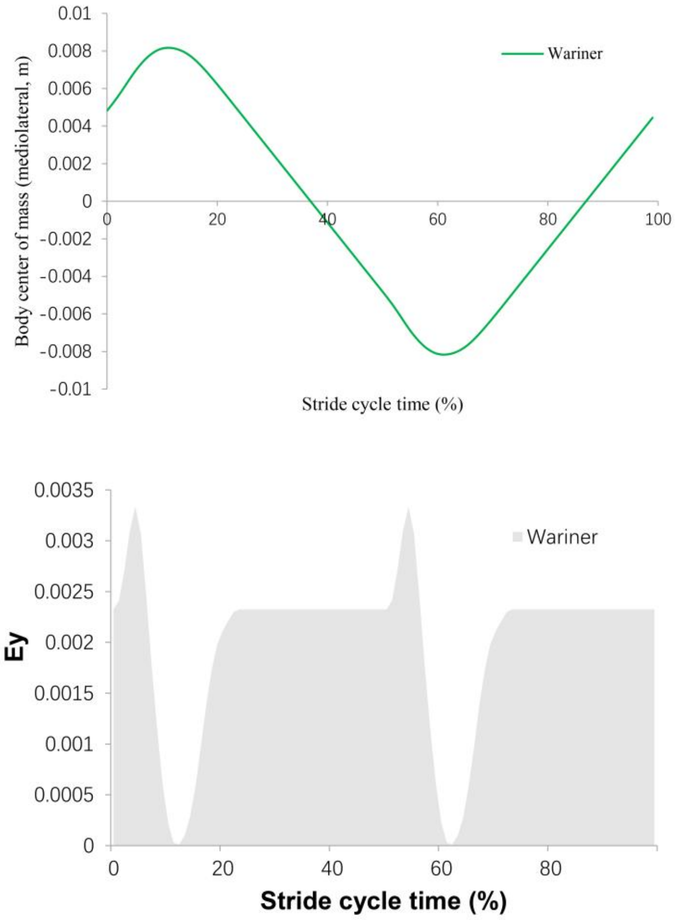

Figure S7. Mediolateral COM displacement and mechanical energy-related variable derived from Wariner's biomechanical data.

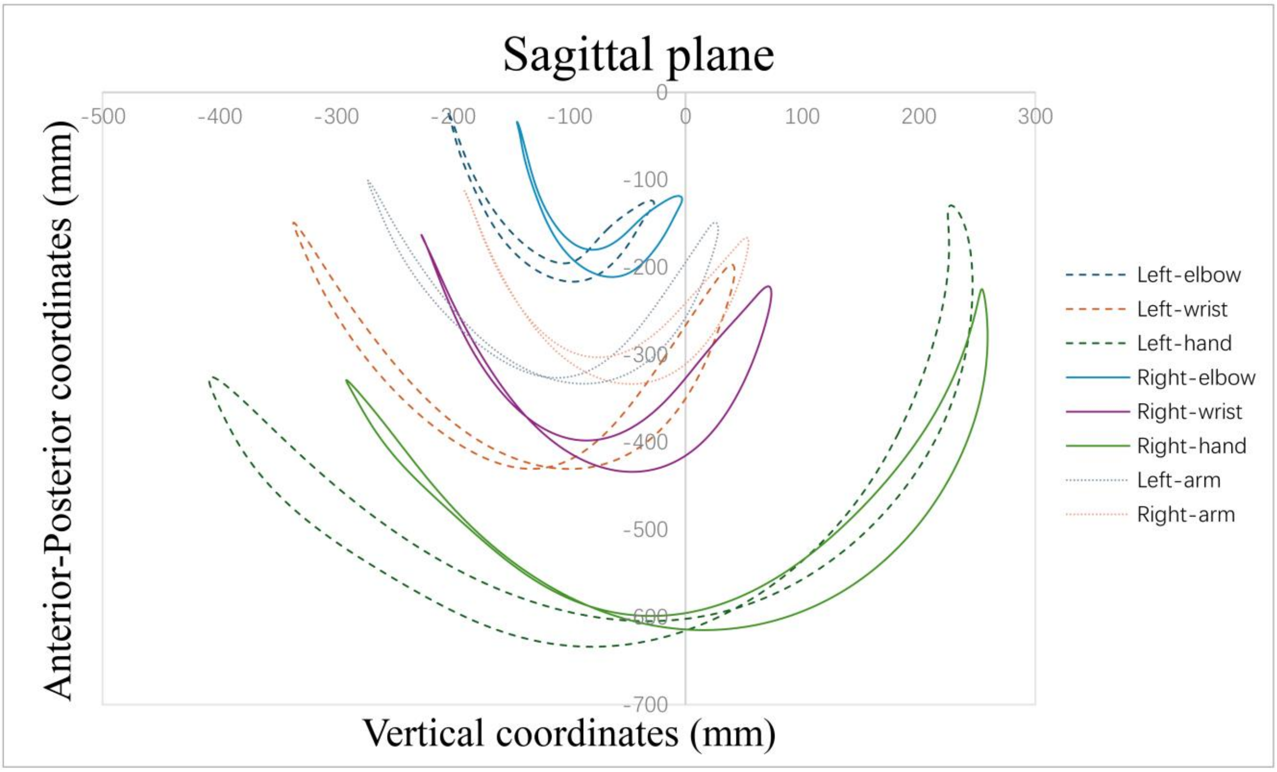

Figure S8. Sagittal-plane upper-limb trajectory relative to the shoulder joint. The upper-limb landmarks and segmental trajectories were analyzed using the shoulder joint/acromion process as the reference point.

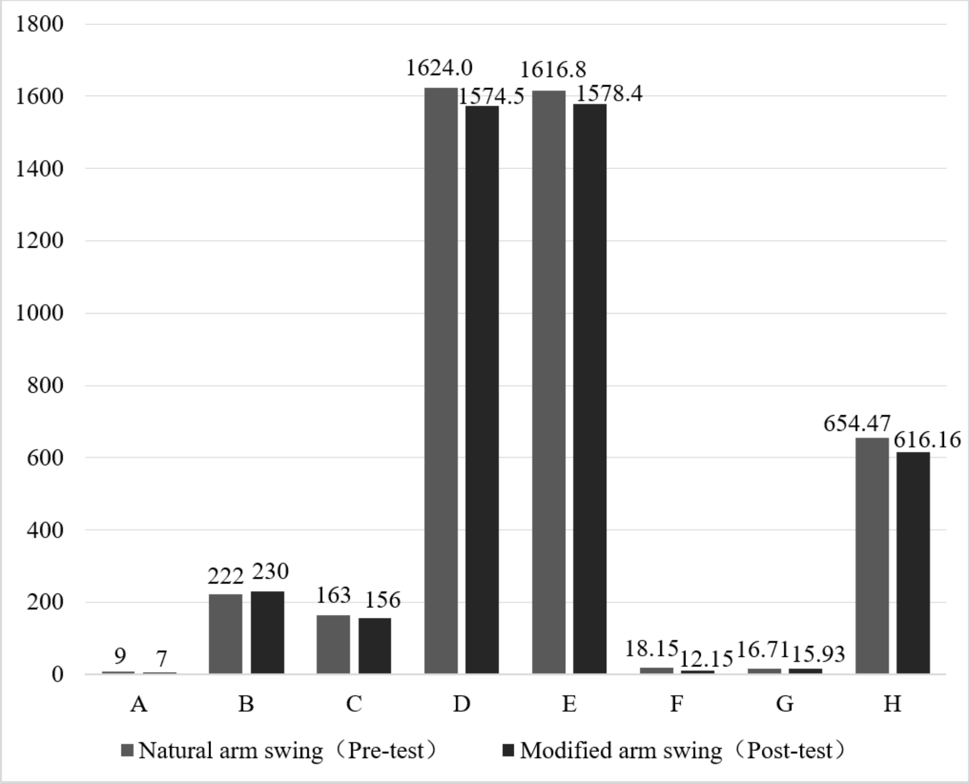

Figure S9 The gait and GRF parameters between natural (pre-test) and modified (post-test) arm swing. Gray bars indicate natural (pre-test); black bars indicate modified (post-test). Parameters shown are: (A) Step width. (B) Step length. (C) Cadence. (D) Maximum force L. (E) Maximum force R. (F) Arm contribution to forward drive (%). (G) Arm contribution to vertical lift (%). (H) Energy consumption. Numerical values are shown above each bar for comparison.

Table S1 Estimation of energy expenditure of running calculated by common equations in physiology.

Equation	VO_2 ($ml \cdot kg^{-1} \cdot min$)	Energy (kJ)	Energy (cal)
ACSM (American College of Sports Medicine, 2013)	39.50	3334.59	796.99
Weyand (Weyand et al., 2021)	30.27	2555.39	610.75
Pandolf (Pandolf et al., 1977)	44.78	3779.91	903.42
Van der Walt (van der Walt and Wyndham, 1973)	27.64	2333.14	557.63
Lejeune (Lejeune et al., 1998)	36.37	3070.58	733.89
